# Random-PE: an efficient integration of random sequences into mammalian genome by prime editing

**DOI:** 10.1186/s43556-021-00057-w

**Published:** 2021-11-18

**Authors:** Yaoge Jiao, Lifang Zhou, Rui Tao, Yanhong Wang, Yun Hu, Lurong Jiang, Li Li, Shaohua Yao

**Affiliations:** State Key Laboratory of Biotherapy, Laboratory of Biotherapy, National Key Laboratory of Biotherapy, Cancer Center, West China Hospital, Sichuan University, Renmin Nanlu 17, Chengdu, 610041 Sichuan China

**Keywords:** CRISPR/Cas9, Prime editing, Random sequence, Gene evolution

## Abstract

Prime editing (PE) enables efficiently targeted introduction of multiple types of small-sized genetic change without requiring double-strand breaks or donor templates. Here we designed a simple strategy to introduce random DNA sequences into targeted genomic loci by prime editing, which we named random prime editing (Random-PE). In our strategy, the prime editing guide RNA (pegRNA) was engineered to harbor random sequences between the primer binding sequence (PBS) and homologous arm (HA) of the reverse transcriptase templates. With these pegRNAs, we achieved efficient targeted insertion or substitution of random sequences with different lengths, ranging from 5 to 10, in mammalian cells. Importantly, the diversity of inserted sequences is well preserved. By fine-tuning the design of random sequences, we were able to make simultaneously insertions or substitutions of random sequences in multiple sites, allowing in situ evolution of multiple positions in a given protein. Therefore, these results provide a framework for targeted integration of random sequences into genomes, which can be redirected for manifold applications, such as in situ protospacer adjacent motif (PAM) library construction, enhancer screening, and DNA barcoding.

**Supplementary Information:**

The online version contains supplementary material available at 10.1186/s43556-021-00057-w.

## Introduction

The genome editing tools based on Clustered regularly interspaced palindromic repeats (CRISPR) Cas9 have shown significant successes in basic biomedical research and also provided great promise in clinical translation [1–3]. Very recently, the development of prime editing technique enables targeted introduction of multiple types of small-sized genetic change in the genome, including deletion, insertion, and base substitution, in an efficient and irreversible way [4]. Meanwhile, this technique does not require the generation of double-strand breaks within the target site, nor does it require donor templates [4]. These features of PE substantially expand the scope and capacity of genome editing, showing great potentials in a large variety of implications [5–8].

Prime editors are composed of a Cas9 and reverse transcriptase domain (RT) fusion protein, a pegRNA and for many cases a nick sgRNA (PE3) [4]. PegRNA is the soul of the PE system in that it not only guides the Cas9 and RT fusion protein (PE2) to the target site to produce a nick in the edited strand, but also provides the nicked DNA with primer binding sequence (PBS) and RT template for the reverse transcription of the former [4]. The lesion caused by reverse transcription of the edited strand will be fixed by endogenous DNA repair or replication mechanisms [9–12] in favor of integrating the desired edits into the genome if a proper homologous arm (HA) is present at the end of the RT-template. Therefore, by fine-tuning the design of pegRNA, prime editing can achieve really re-writing the genome.

Here we designed a simple strategy, named Random-PE, based on prime editing to introduce random sequences into the target region of the mammalian genome. In the Random-PE strategy, the pegRNAs were designed to harbor random sequences in-between the PBS and HA of the reverse transcriptase templates. We showed that the Random-PE strategy achieved efficient targeted insertion or substitution of random sequences up to 10 base pairs (bps), a theoretical diversity of ~ 10^7^. To the best of our knowledge, this is the first attempt of trying to introduce random sequences into mammalian cells using pooled pegRNA library. During the preparation of the manuscript, a similar strategy using plasmid pegRNA library for random prime editing was developed in plant showing that 3-bp random sequences were able to be integrated into rice genome [13]. Together with the result in plant, these works provide a framework for targeted integration of random sequences into the genomes, which can be redirected for manifold applications, such as in situ PAM library construction, enhancer screening, and DNA barcoding.

## Results

### Design of Random-PE

**Fig. 1:**
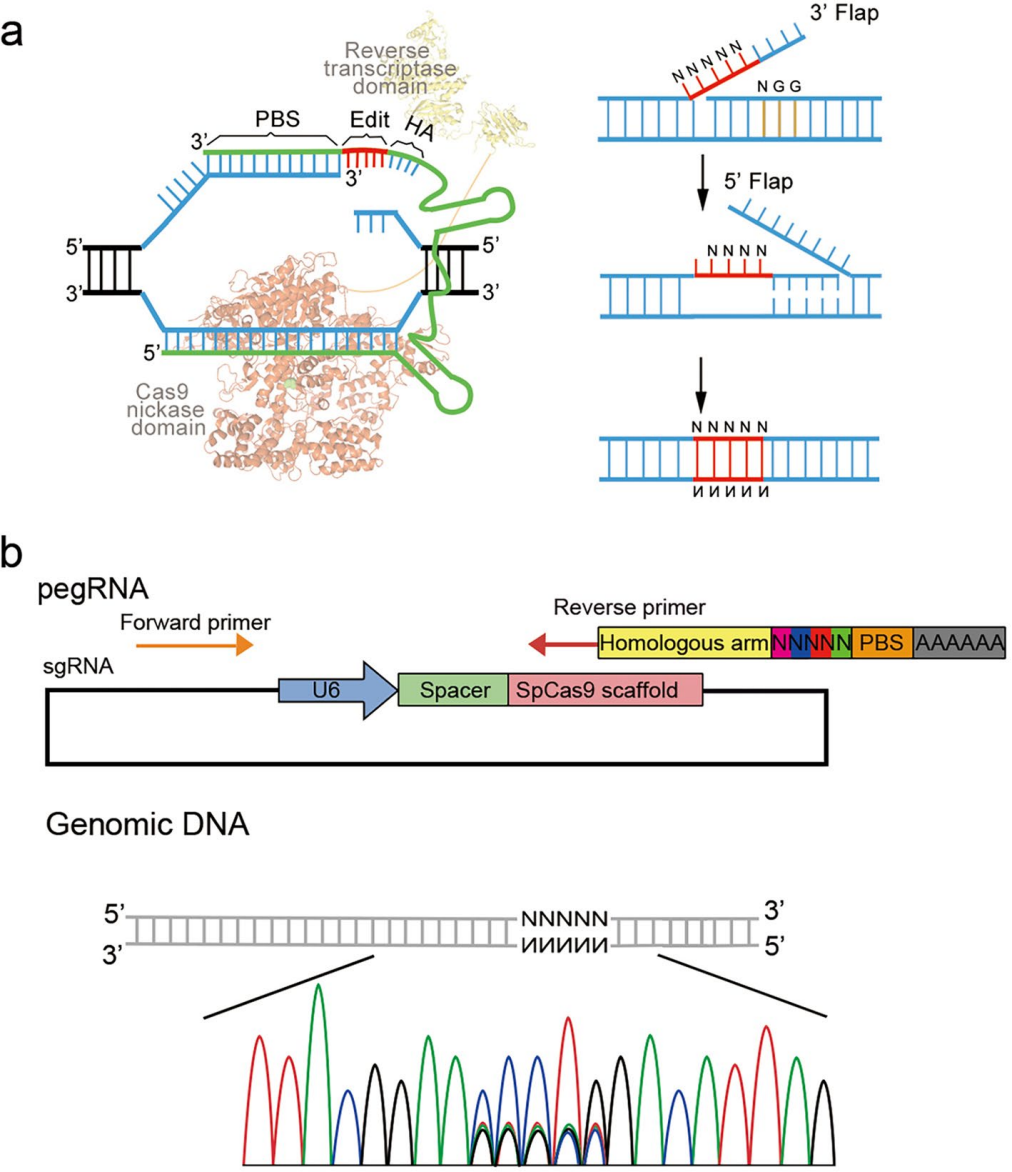
Design of Random-PE. **a**. Diagram showing the design and action of Random-PE. Left panel showed the action of PE, and right panel showed the putative process of the integration of PE-induced edits containing random sequences. **b**. Diagram showing the organization of pegRNA library (upper panel) and the outcomes of Random-PE (lower panel). Note that the cartoon in the lower panel was drawn by photoshop, illustrating Sanger sequencing chromograph of the genomic DNA undergoing Random-PE

The fact that PE can induce targeted substitution, deletion or insertion of small genomic fragments intrigues us to test if we can introduce random sequences into mammalian genomes, thereby enabling in situ targeted evaluation of aimed genes. We designed a strategy, named Random-PE, for such a purpose, in which a library of pegRNAs was engineered to contain PBS, HA, and random sequences in-between them (Fig. 1a). Delivery of the pegRNA library together with the other PE components, including PE2 and nick sgRNA, to mammalian cells or zygotes are supposed to introduce random sequences into the target strand of the aimed loci. Each sequence is then integrated into the genome through HA-mediated DNA repair or replication mechanisms. The diversity of editing events, i.e. the diversity of random sequences integrated into the genome, was therefore correlated with the size of the library and the copy number of genomes to be edited.

As a matter of fact, the construction of pegRNA library containing random sequences is a key parameter for the action of Random-PE. We planned to construct a PCR product library of pegRNAs so that the variety of the library was extensively preserved. In addition, the PCR product library also avoids time-consuming process of plasmid library construction. As a first step to testing the feasibility of PCR library, we investigated if pegRNA, in the form of PCR product, was functional in mammalian cells. We made PCR products of a previously reported pegRNA [4] (DNMT1 G to C) and compared its activity to that of pegRNA plasmids in HEK293T cells (Supplementary Fig. 1a). Notably, the PCR product included U6 promoter, pegRNA, and polymerase 3 termination signal (6 × T). As shown in Supplementary Fig. 1b, PCR products were as efficient as plasmids in PE3 mediated G to C conversions (PCR pegRNA VS plasmid pegRNA = 12%: 14%).

### Introducing random sequences into mammalian genome via random-PE

**Fig. 2:**
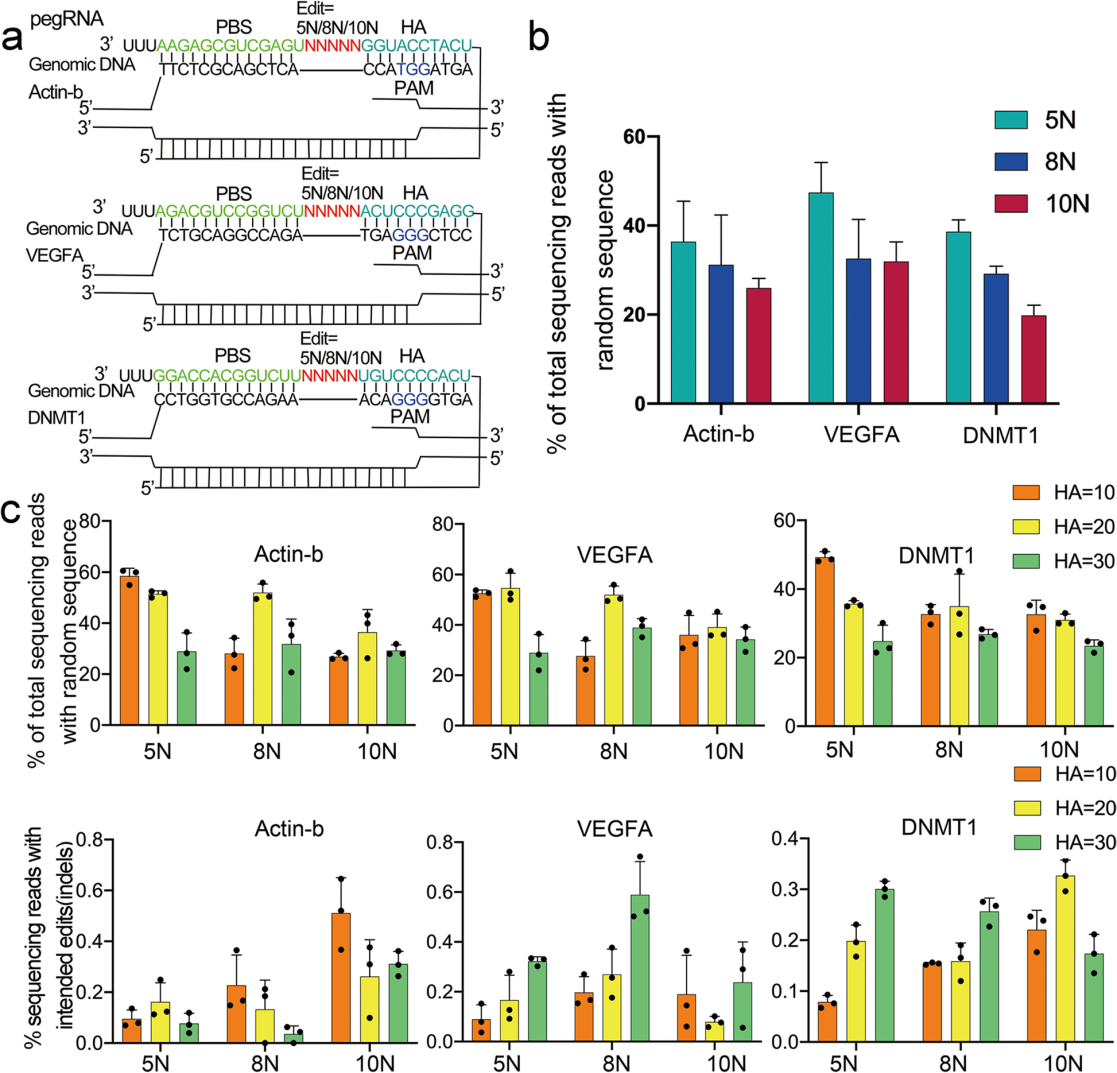
Introducing random sequences into mammalian genomes by Random-PE. **a**. Diagram showing the design of pegRNAs targeting Actin-b, VEGFA and DNMT1 loci. PBS was shown in green; HA was shown in cyan and the random sequence was shown in red. **b**. Targeted insertions of 5 bp, 8 bp and 10 bp random sequences in Actin-b, VEGFA and DNMT1 loci (PBS = 13 nt, HA = 10 nt). **c**. Optimization of the HA length in different editing events. Upper panels showed the efficiencies of targeted insertions of 5 bp, 8 bp and 10 bp random sequences in Actin-b, VEGFA, and DNMT1 loci at indicated HA lengths, and lower panels showed the level of undesired indels accompanied with the insertions. Values and error bars reflect mean ± s .d. of *n* = 3 independent biological replicates

Intrigued by this result, we constructed a pegRNA library targeting DNMT1, which contained a piece of 5-bp random sequence flanked by 13 nt PBS and 10 nt HA (Fig. 2a). This pair of PBS and HA had been previously verified to be able to induce efficient prime editing at the site of ssDNA break of the edited strand [4]. The library was generated by PCR amplification of the existing DNMT1 sgRNA as depicted in Fig. 1b. Transfection of the DNMT1 pegRNA library together with PE2 and DNMT1 nick sgRNA into HEK293T cells did produce a considerable level of targeted insertion of random sequences, as evidenced by Sanger sequencing (Supplementary Fig. 2). In the sequencing chromatogram, a 5 bp fragment containing multiple traces of all four colors occurred at the aimed position (3-bp upstream the NGG PAM), indicating successfully targeted insertion of random sequences. Consistently, double peaks occurred following the 5 bp random sequences, in which both peaks were ascribed to wildtype DNMT1 genomic sequence with lower peaks 5 bp proceeding higher peaks, a sign of 5 bp insertion. Therefore, these results demonstrated the feasibility of PCR pegRNA library in Random-PE.

To confirm the universality of our PCR-based library, we tested the strategy at additional two endogenous loci, Actin-b and VEGFA. Similar to the conditions in the DNMT1 pegRNA library, Actin-b and VEGFA libraries were designed to harbor a piece of 5-bp random sequences flanked by 13 nt PBS and 10 nt HA (Fig. 2a). Transfection of the two libraries together with PE2 and corresponding nick sgRNAs into HEK293T cells produced obvious targeted insertion of random sequences, as characterized by key signs in Sanger sequencing chromatograms (Supplementary Fig. 2). To quantify the editing efficiency, we performed high-throughput sequencing and found robust editing across all three targeted inserts (Actin-b = 36.35 ± 9.11%, VEGFA = 47.45 ± 6.71%, DNMTI = 38.64 ± 2.63%, Fig. 2b). Next, we tested if random sequences longer than 5 bp was able to be inserted into the genome by Random-PE. We extended the length of random sequences to 8 bp or 10 bp while kept PBS and HA intact. As shown in Fig. 2b, all these libraries induced efficient insertion of random sequences. For the insertion of 8 bp random sequences, the average editing efficiency of Actin-b, VEGFA and DNMT1 were 31.39 ± 11.20%, 32.56 ± 8.84% and 29.17 ± 1.73% respectively. The average editing efficiency of 10 bp random sequences in each locus was slightly lower than that of 8 bp random sequences (Actin-b = 25.98 ± 2.16%, VEGFA = 31.91 ± 4.41% and DNMT1 = 19.81 ± 2.30%). Taken together, these results demonstrated the universality of Random-PE. Although we did not test longer random sequences, previous PE experiments in targeted insertion of loxP site suggested that the maximum length of Random-PE would exceed 40 bp [4].

### The effects of the HA length on editing efficiency of random-PE

It has been observed that the length of HA is a key parameter determining the efficiency of PE. To investigate the optimized length of HA in each insertion, we extended the length of HA from 10 nt to 20 nt and 30 nt respectively, while kept PBS intact. Consistent with previous studies [4], the optimal HA length was found to vary with individual target sites and the length of insertions. At Actin-b loci, the optimal HA length was 10 nt for 5 bp insertion and 20 nt for 8 bp and 10 bp insertions. At DNMT1 loci, the optimal HA length was 10 nt for 5 bp and 10 bp insertions and 20 nt for 8 bp insertion. And the optimal HA length was 10 nt for all insertions at VEGFA loci. Notably, the efficiencies of 10 nt and 20 nt were comparable across most editing events, the level of which was much higher than that of 30 nt (Fig. 2c). Given that lengthening the HA will burden the construction process of pegRNA library, we recommend starting with 10 nt HA during the optimization of pegRNA library. Analysis of the HTS data revealed that the level of undesired indel was minimal across all events, with the highest one being 0.59 ± 0.13% (Fig. 2c).

### In vivo gene evolution via random-PE

**Fig. 3:**
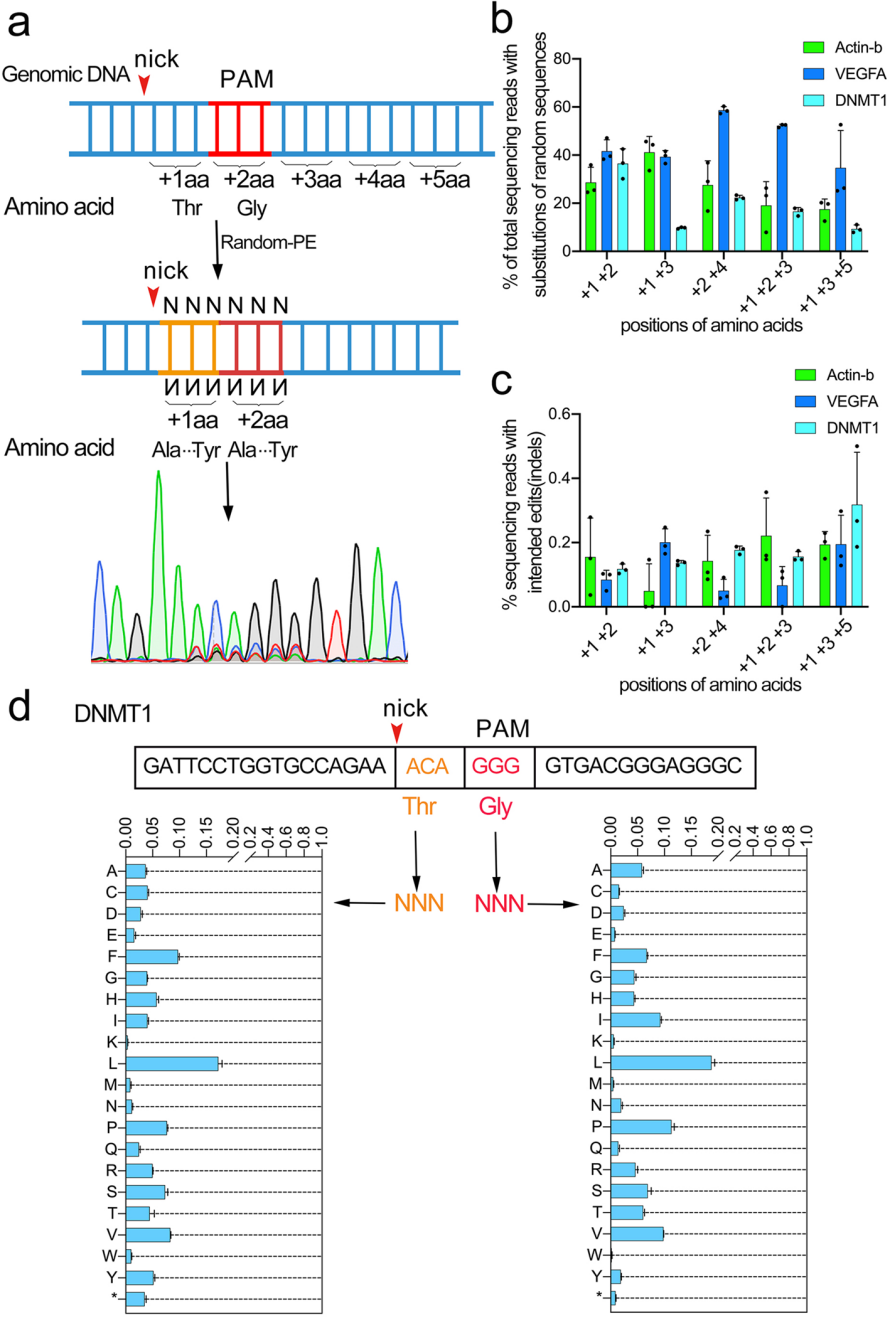
Random-PE produced unbiased amino acid substitutions in endogenous genes. **a**. Diagram showing the design and nomination of Random-PE induced unbiased amino acid substitutions. The red arrow head indicated the nick in the edited strands produced by Cas9 nickase. PAM was shown in red. The first triplet codon 3’adjacent to the nick was nominated as +1aa. **b**. Efficiencies of unbiased amino acid substitutions induced by Random-PE at different positions of Actin-b, VEGFA and DNMT1 genes. **c**. Undesired indels induced by Random-PE during unbiased amino acid substitutions. **d**. Distribution of different amino acids in the Random-PE mediated substitution. * represents the stop codon. Values and error bars reflect mean ± s.d. of n = 3 independent biological replicates

After establishing that our Random-PE was efficient in introducing random sequences into the mammalian genome, we further examined if Random-PE could be rewired for the purpose of unbiased gene evolution [13, 14]. To this end, we imitated the evolution by mutating two or three amino acids within Actin-b, VEGFA and DNMTI genes. We nominated the triplet codon 3’adjacent to the nick of the edited strand as the first amino acid (+1aa) (Fig. 3a). We started the imitation at DNMTI gene, in which both +1aa and + 2aa were designed to harbor random mutations. As shown in the sanger sequencing chromatograph of Fig. 3a, a considerable portion of +1aa (Threonine) and + 2aa(Glycine) were substituted by random amino acids, suggesting successful random mutation. We quantified the level of substitution by HTS and found 36.47% of alleles were mutated. We then extended such design to Actin-b and VEGFA genes and found comparable levels of desired substitutions to that from DNMT1 genes. To expand the scope of targeted evolution, we design additional types of substitutions, including +1aa + 3aa, +2aa + 4aa, +1aa + 2aa + 3aa, +1aa + 3aa + 5aa. As shown in Fig. 3b, all five types produced obvious substitutions across 3 target genes as determined by HTS, albeit their efficiencies varying with the substitution types and genes. The analysis of the HTS data revealed that the level of undesired indel was minimal across all substitution events, with the highest one being 0.22 ± 0.12% (Fig. 3c). Moreover, an examination of the amino acids encoded by the random substitutions identified all 21 types of amino acids as well as the stop codon. However, the distributions of these codons were not even (Supplementary Fig. 3, 4 and 5). Taken +1aa + 2aa substitution in DNMT1 as an example, the frequencies of individual codons ranged from 0.20 to 18.78%. The maximal frequency occurred at the codon of leucine (17.21% for+1aa and 18.78% for +2aa) and minimal ones occurred at Tryptophan and Lysine(+1aa Tryptophan 1.01%; +2aa Tryptophan 0.20%; +1aa Lysine 0.36%; +2aa Lysine 0.58%)(Fig. 3d). This phenomenon was possibly due to that the distributions of each random sequence in the library or their editing efficiencies were inhomogeneous and that the amino acid codons themselves were inhomogeneous.

### The workflow of random-PE in mammalian cells

**Fig. 4:**
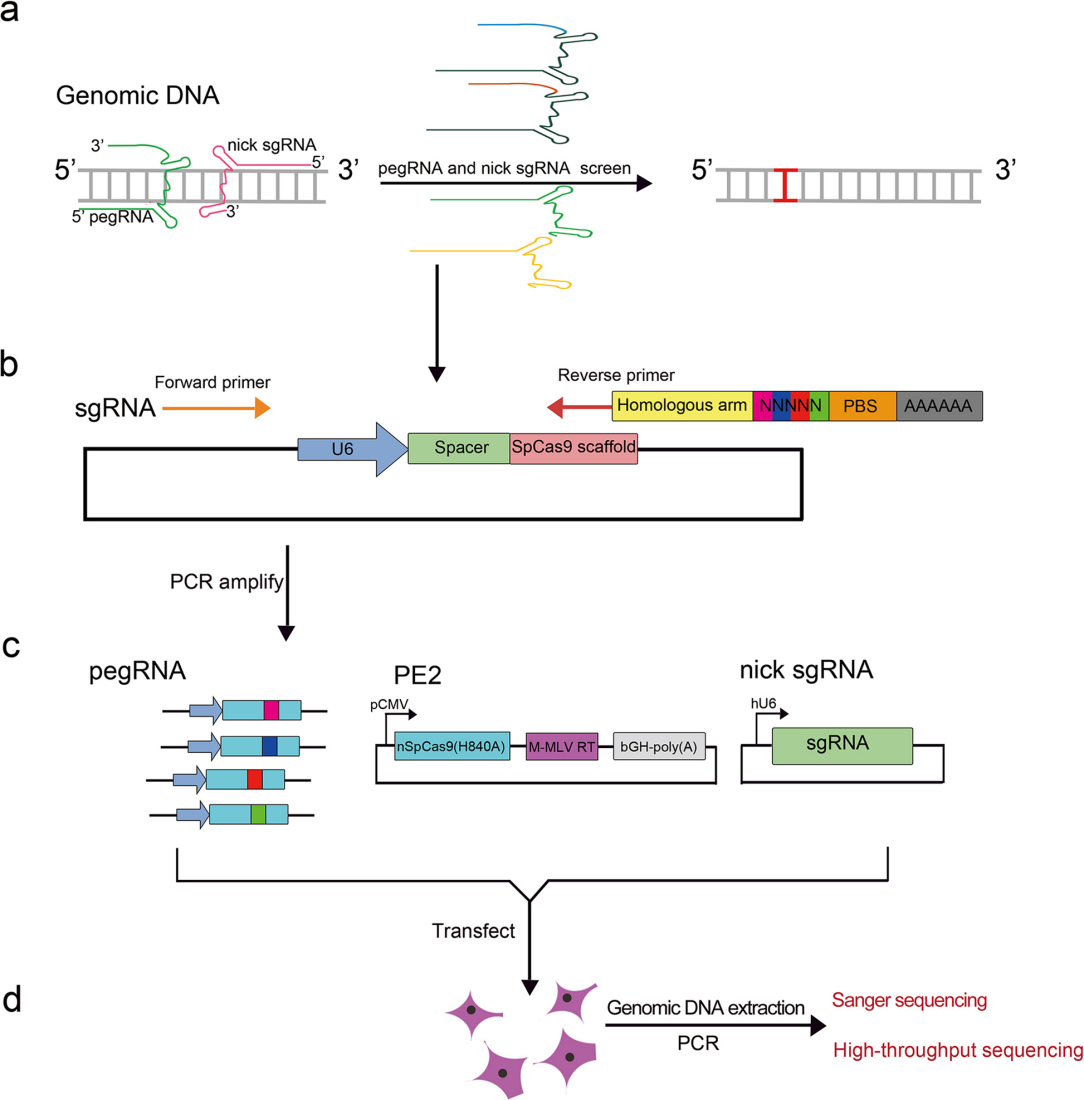
Workflow of Random-PE in cultured mammalian cells. **a**. Diagram showing the design and screen of functional PE3 system targeting interest genes. **b**. Construction of PCR pegRNA random library. A forward primer located upstream of U6 promoter and a reverse primer containing complementary sequences to the sgRNA scaffold, HA, aimed random sequences, PBS, and U6 termination signal are used to amplify the existing sgRNA or pegRNA. **c**. Delivery of pegRNA library together with PE2 and nick sgRNA plasmids into interest cells. **d**. Detection of the desired insertion of random sequences in the genome. Genomic DNAs are extracted from transfected cells and subjected to PCR amplification using primers flanking the target region. The presence of the desired insertion of random sequences is detected by Sanger sequencing and quantified by HTS

The above results demonstrated the feasibility of our Random-PE strategy in targeted integration of random sequences into mammalian genomes. To gain an insight into the practical performance of this strategy, we summarized the workflow of relevant experimental processes. For a given aimed editing, the first step is to establish a functional PE3 for the target. Single base conversion PE3 is a good choice because its editing outcomes can be evaluated simply by Sanger sequencing (Fig. 4a). The parameters of pegRNA, mainly the position of edits (relative to the PAM), the length of PBS and HA, and the position of nick sgRNA, should follow basic rules raised by previous literature [4, 15–17]. After establishing a functional PE3, optimization of PBS, HA, and nick sgRNA may be helpful to improve the editing efficiency. Next, the pegRNA library containing aimed random sequences are prepared by normal PCR protocol using paired primers flanking the necessary elements for the expression and function of pegRNA. Noteworthy, as elucidated in Fig. 4b, the reverse primer encodes polyT termination signal, PBS, random sequences, HA, and a fragment complementary to the 3’end of the scaffold. The quality of the PCR amplified pegRNA library should be verified by methods such as agarose gel or capillary electrophoresis. Normally, the amplification generates homogeneous products. The products are purified by PCR purification kits to remove unnecessary impurities that might interfere with cell transfection (Fig. 4c). Then the PCR library is co-transfected into interested cells together with PE2 and nick sgRNA plasmids. DNA extraction and PCR analysis are performed after 2–3 days of action of the PE system. The presence of random sequences in the genome can be detected by Sanger sequencing and then quantified by HTS (Fig. 4d).

## Discussion

In summary, we have developed a prime editing strategy, Random-PE, to introduce random sequences into mammalian genomes. We showed that Random-PE achieved targeted integration of up to 10 bp random sequence (diversity of the library > 10^7) at an efficiency of up to 39.07%. According to previous PE experiments in targeted insertion, the maximum length of Random-PE would exceed 40 bp, a diversity of 10^24^ [4]. We believe that our Random-PE strategy will have potentials in a wide range of amplifications such as in situ barcoding, PAM library, and unbiased gene evolution, etc.

In the attempt to perform in vivo gene evolution, we noticed that the proverbiality of individual triplet installed was not even. This phenomenon was possibly due to that the distributions of each random sequence in the library or their editing efficiencies were inhomogeneous. Previous study has revealed that efficiencies of different editing evens might varied even they share common key features, including spacer, PBS and HA of the pegRNA, all of which are known to determine the prime editing efficiency [4]. For example, in prime editing medicated single base conversions of RUNX1 site (+ 1), C to G, and C to T conversions were ~ 2 times more efficient than that of C to A. In addition, it has been shown that 3′ extension of pegRNA, in particular the PBS, is capable of base-pairing with the spacer, leading to reduced prime editing [18]. Therefore, it is also possible that specific triplets in the 3′ extensions may disturb the architecture of pegRNA, thereby reducing prime editing.

Recently, several progresses have been made in improving the efficiency of prime editing. Equipping the prime editor with strong nuclear localization signal [19], 3′ engineered pegRNA [18, 20] or ssDNA binding domain [21] significantly enhanced prime editing. Moreover, endogenous mismatch repair pathway has been shown to inhibit the installation of the desired edits, and depletion of this pathway also improved prime editing [22]. Therefore, it is reasonable to hypothesize that these progresses may be introduced to Random-PE to enhance its efficiency.

## Materials and methods

### Construction of plasmids and pegRNA library

The plasmid PE2 was obtained from addgene (#132775). The sgRNA plasmids were constructed by ligating annealed oligonucleotide duplexes into pU6 sgRNA cut with Bbs1. Oligos used to generate spacers were listed in Supplementary Table S1. pegRNAs were constructed by PCR-mediated elongation of each sgRNAs, and those used for normal PE were cloned into pESI-Blunt vector (Yeasen Biotechnology). To ensure the diversity of the pegRNA library, a total of 0.02 nmol random primers (about 1 × 10^13^ DNA molecules) were used when performing PCR amplification. The primers used to construct pegRNA were listed in Supplementary Table S4. The sequences of pegRNAs were shown in Supplementary Table S3.

### Cell culture and transfection

HEK293T cells were cultured in DMEM medium (Gibco® by Life Technologies), supplemented with 10% fetal bovine serum (Life Technologies) and 1% Penicillin/Streptomycin (Boster Biological Technology Co. Ltd) at 37 °C and 5% CO2.

HEK293T cells were plated into 96-well plates 12–24 h before transfection, and each well was seeded with 2 × 10^5^ cells. Cells at a confluence of ~ 80% were transfected with plasmids encoding PE2(327 ng, ~ 0.06 pmol) and the pegRNAs in PCR form(36 ng, ~ 0.06 pmol) and plasmids encoding nick sgRNAs(36 ng, ~ 0.02 pmol), with molar ratio of PE2: pegRNA library: nick sgRNA≈ 3:3:1, using Transeasy™ (Forgene).

### Sanger sequencing of genomic DNA samples

Cells were harvested 72 h post-transfection and the genomic DNA was extracted with freshly prepared DNA extraction buffer. Genomic regions of interest were amplified by PCR and then were analyzed with Sanger sequencing. The sequences of primers were listed in Supplementary Table S2. Single base conversion was quantified by EditR software (http://baseditr.com), according to the author’s description [23]. Genomic DNAs with the integrated random sequences were actually the combination of numerous specific sequences that were randomly and individually integrated into the target region; therefore, Sanger sequencing of such region will identify multiple traces of all four bases.

### Deep sequencing of genomic DNA samples and data analysis

Genomic regions of interest were amplified by High-Fidelity DNA Polymerase (Phanta® Max Super-Fidelity) with primers flanked with different barcodes (supplementary Table S5). The PCR products were gel-purified and quantified with Nano Drop (thermo scientific). Samples were sequenced commercially using the Ilumina Novaseq-2000 platform (Personal Biotechnology. Shanghai. China). The frequencies of insertions and substitutions and indels were quantified as the percentage of total sequencing reads. GraphPad Prism 8 software was used to analyze the data. All values are presented as mean ± standard deviation (sd).

## Supplementary Information

**Additional file 1: Supplementary Figure 1.** Comparison of the efficiency of pegRNAs in the form of plasmid and PCR product. **Supplementary Figure 2.** Detecting the activity of Random-PE in Actin-b, VEGFA and DNMT1 loci with Sanger sequencing. **Supplementary Figure 3.** Distribution of various codons in Actin-b gene edited by Random-PE. **Supplementary Figure 4.** Distribution of various codons in VEGFA gene edited by Random-PE. **Supplementary Figure 5.** Distribution of various codons in DNMT1 gene edited by Random-PE. **Supplementary Table 1.** List of the targets tested in this study. **Supplementary Table 2.** Sequences of primers used for mammalian cell genomic DNA amplification. **Supplementary Table 3.** Sequences of pegRNAs used in mammalian cell experiments. **Supplementary Table 4.** Sequences of primers for amplification of pegRNAs. **Supplementary Table 5.** Sequences of primers used for HTS.

## Data Availability

All data generated or used during this study appear in the submitted article and its supplementary files.

## Abbreviation

CRISPR: Clustered Regularly Interspaced Short Palindromic Repeats
HA: Homologous arm
HTS: High-throughput sequencing
PAM: Protospacer adjacent motif
PBS: Primer binding sequence
PCR: Polymerase Chain Reaction
PE: Prime editing
PE2: Prime editor 2
PE3: Prime editor 3 systems
pegRNA: Prime editing guide RNA
RT: Reverse transcriptase
Random-PE: Random prime editing
sgRNA: Small guide RNA
ssDNA: Single-stranded DNA

## References

[1] Dever DP, Bak RO, Reinisch A, Camarena J, Washington G, Nicolas CE (2016). CRISPR/Cas9 beta-globin gene targeting in human haematopoietic stem cells. Nature..

[2] Mettananda S, Fisher CA, Hay D, Badat M, Quek L, Clark K (2017). Editing an alpha-globin enhancer in primary human hematopoietic stem cells as a treatment for beta-thalassemia. Nat Commun.

[3] Nelson CE, Hakim CH, Ousterout DG, Thakore PI, Moreb EA, Castellanos Rivera RM (2016). In vivo genome editing improves muscle function in a mouse model of Duchenne muscular dystrophy. Science..

[4] Anzalone AV, Randolph PB, Davis JR, Sousa AA, Koblan LW, Levy JM (2019). Search-and-replace genome editing without double-strand breaks or donor DNA. Nature..

[5] Schene IF, Joore IP, Oka R, Mokry M, van Vugt AHM, van Boxtel R (2020). Prime editing for functional repair in patient-derived disease models. Nat Commun.

[6] Liu Y, Li X, He S, Huang S, Li C, Chen Y, et al. Efficient generation of mouse models with the prime editing system. Cell Discov. 2020;627 10.1038/s41421-020-0165-z. 10.1038/s41421-020-0165-z PMC7186222 32351707

[7] Lin Q, Zong Y, Xue C, Wang S, Jin S, Zhu Z (2020). Prime genome editing in rice and wheat. Nat Biotechnol.

[8] Kim DY, Moon SB, Ko JH, Kim YS, Kim D (2020). Unbiased investigation of specificities of prime editing systems in human cells. Nucleic Acids Res.

[9] Liu Y, Kao HI, Bambara RA. Flap endonuclease 1: a central component of DNA metabolism. Annu Rev Biochem. 2004:73589–615 10.1146/annurev.biochem.73.012803.092453. 10.1146/annurev.biochem.73.012803.092453 15189154

[10] Kosicki M, Tomberg K, Bradley A (2018). Repair of double-strand breaks induced by CRISPR-Cas9 leads to large deletions and complex rearrangements. Nat Biotechnol.

[11] Hanscom T, McVey M. Regulation of Error-Prone DNA Double-Strand Break Repair and Its Impact on Genome Evolution. Cells. 2020; 9(7).10.3390/cells9071657. 10.3390/cells9071657 PMC7407515 32660124

[12] Ceccaldi R, Rondinelli B, D'Andrea AD (2016). Repair pathway choices and consequences at the double-Strand break. Trends Cell Biol.

[13] Xu R, Liu X, Li J, Qin R, Wei P (2021). Identification of herbicide resistance OsACC1 mutations via in planta prime-editing-library screening in rice. Nat Plants.

[14] Roy KR, Smith JD, Vonesch SC, Lin G, Tu CS, Lederer AR (2018). Multiplexed precision genome editing with trackable genomic barcodes in yeast. Nat Biotechnol.

[15] Anderson MV, Haldrup J, Thomsen EA, Wolff JH, Mikkelsen JG (2021). pegIT - a web-based design tool for prime editing. Nucleic Acids Res.

[16] Hsu JY, Grunewald J, Szalay R, Shih J, Anzalone AV, Lam KC (2021). PrimeDesign software for rapid and simplified design of prime editing guide RNAs. Nat Commun.

[17] Hwang GH, Jeong YK, Habib O, Hong SA, Lim K, Kim JS (2021). PE-designer and PE-analyzer: web-based design and analysis tools for CRISPR prime editing. Nucleic Acids Res.

[18] Liu Y, Yang G, Huang S, Li X, Wang X, Li G (2021). Enhancing prime editing by Csy4-mediated processing of pegRNA. Cell Res.

[19] Liu P, Liang SQ, Zheng C, Mintzer E, Zhao YG, Ponnienselvan K (2021). Improved prime editors enable pathogenic allele correction and cancer modelling in adult mice. Nat Commun.

[20] Nelson JW, Randolph PB, Shen SP, Everette KA, Chen PJ, Anzalone AV, et al. Engineered pegRNAs improve prime editing efficiency. Nat Biotechnol. 2021. 10.1038/s41587-021-01039-7. 10.1038/s41587-021-01039-7 PMC8930418 34608327

[21] Song M, Lim JM, Min S, Oh JS, Kim DY, Woo JS (2021). Generation of a more efficient prime editor 2 by addition of the Rad51 DNA-binding domain. Nat Commun.

[22] Chen PJ, Hussmann JA, Yan J, Knipping F, Ravisankar P, Chen PF, et al. Enhanced prime editing systems by manipulating cellular determinants of editing outcomes. Cell. 2021. 10.1016/j.cell.2021.09.018. 10.1016/j.cell.2021.09.018 PMC8584034 34653350

[23] Kluesner MG, Nedveck DA, Lahr WS, Garbe JR, Abrahante JE, Webber BR, et al. EditR: a method to Quantify Base editing from sanger sequencing. CRISPR J. 2018:1239–50 10.1089/crispr.2018.0014. 10.1089/crispr.2018.0014 PMC6694769 31021262

