# Supplementary material for: Random-PE: an efficient integration of random sequences into mammalian genome by prime editing
Source: Mol Biomed. 2021 Nov 18;2:36. doi: 10.1186/s43556-021-00057-w (PMC8607425; doi:10.1186/s43556-021-00057-w)
Supplement: Supplementary file 1 — Additional file 1: Supplementary Figure 1. Comparison of the efficiency of pegRNAs in the form of plasmid and PCR product. Supplementary Figure 2. Detecting the activity of Random-PE in Actin-b, VEGFA and DNMT1 loci with Sanger sequencing. Supplementary Figure 3. Distribution of various codons in Actin-b gene edited by Random-PE. Supplementary Figure 4. Distribution of various codons in VEGFA gene edited by Random-PE. Supplementary Figure 5. Distribution of various codons in DNMT1 gene edited by Random-PE. Supplementary Table 1. List of the targets tested in this study. Supplementary Table 2. Sequences of primers used for mammalian cell genomic DNA amplification. Supplementary Table 3. Sequences of pegRNAs used in mammalian cell experiments. Supplementary Table 4. Sequences of primers for amplification of pegRNAs. Supplementary Table 5. Sequences of primers used for HTS. [file 43556_2021_57_MOESM1_ESM.docx]

**Supplementary information**

**Random-PE：an efficient integration of random sequences into mammalian genome by prime editing**

**Contents**

Supplementary Figure 1. Comparison of the efficiency of pegRNAs in the form of plasmid and PCR product.

Supplementary Figure 2. Detecting the activity of Random-PE in Actin-b, VEGFA and DNMT1 loci with Sanger sequencing.

Supplementary Figure 3. Distribution of various codons in Actin-b gene edited by Random-PE.

Supplementary Figure 4. Distribution of various codons in VEGFA gene edited by Random-PE.

Supplementary Figure 5. Distribution of various codons in DNMT1 gene edited by Random-PE.

Supplementary Table 1. List of the targets tested in this study.

Supplementary Table 2. Sequences of primers used for mammalian cell genomic DNA amplification.

Supplementary Table 3. Sequences of pegRNAs used in mammalian cell experiments.

Supplementary Table 4. Sequences of primers for amplification of pegRNAs.

Supplementary Table 5. Sequences of primers used for HTS.


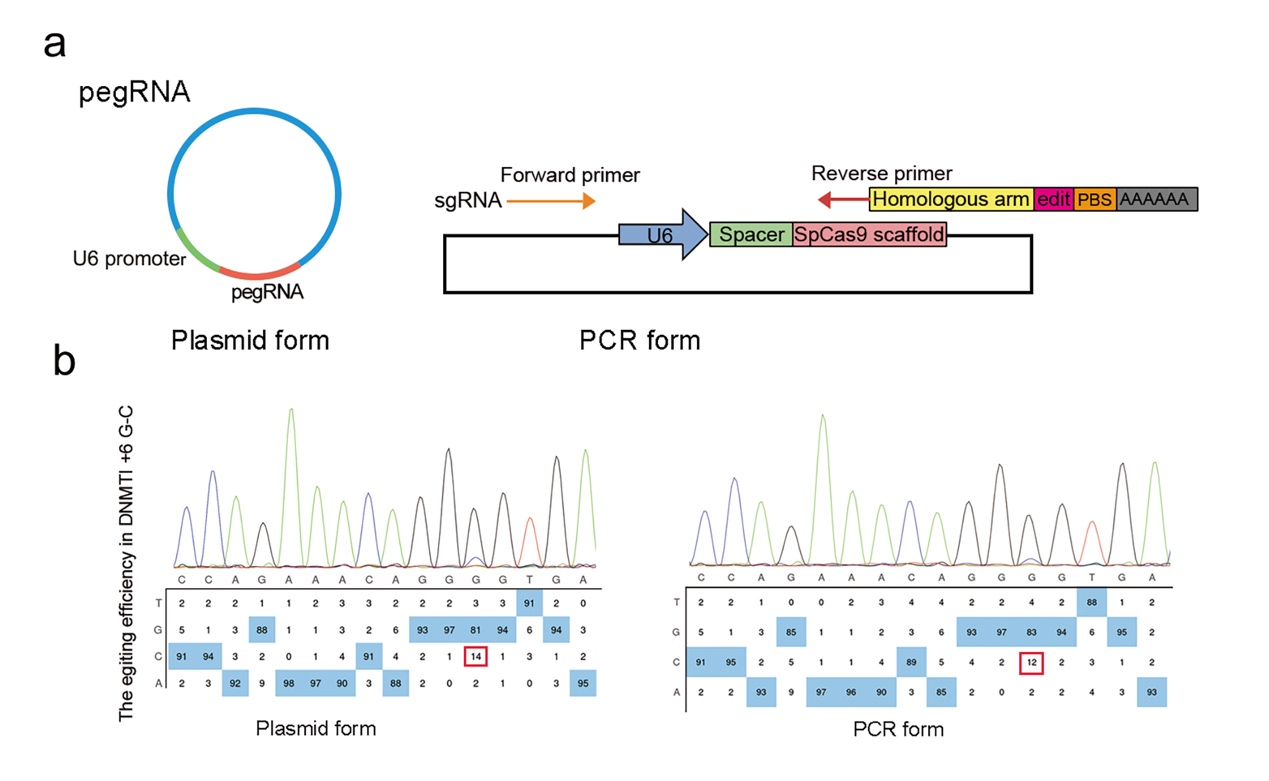


**Supplementary Figure 1.** Comparison of the efficiency of pegRNAs in the form of plasmid and PCR product.

a. Diagram showing pegRNAs in the form of plasmid and PCR product. b. Representative Sanger sequencing results of PE3 mediated G to C conversion in DNMT1 locus. The level of conversion induced by PCR pegRNA was comparable to that by plasmid pegRNA.


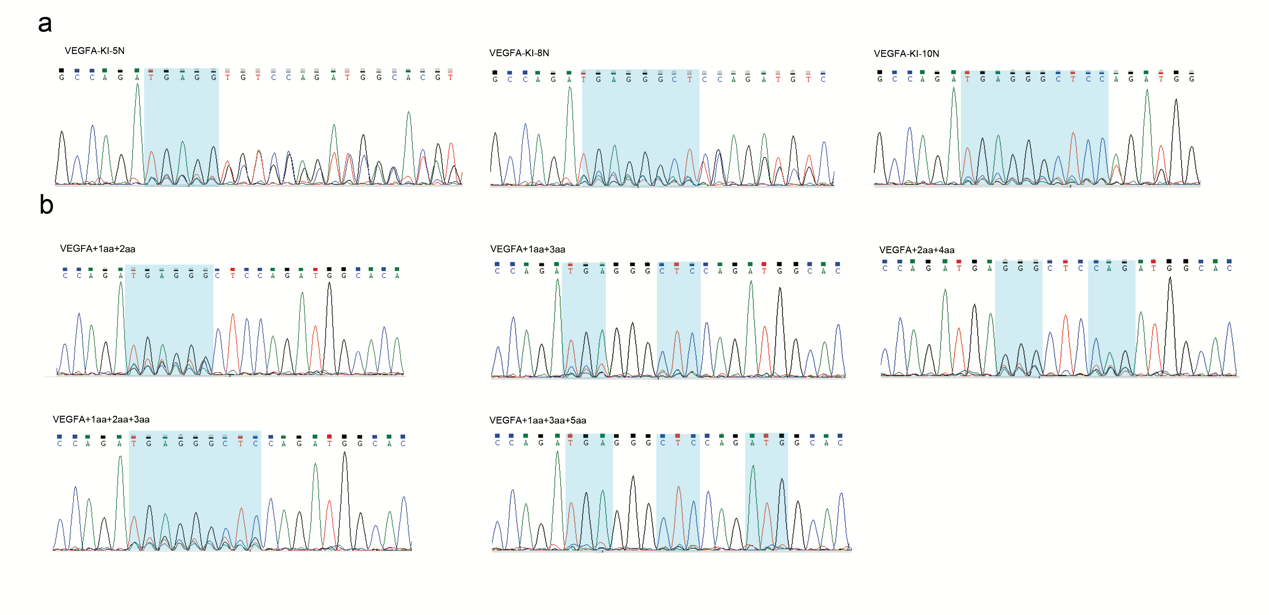


**Supplementary Figure 2.** Detecting the activity of Random-PE in Actin-b, VEGFA and DNMT1 loci with Sanger sequencing.

Representative Sanger sequencing results of Random-PE mediated insertion of 5 bp, 8bp and 10bp random sequences in Actin-b, VEGFA and DNMT1 genes respectively.


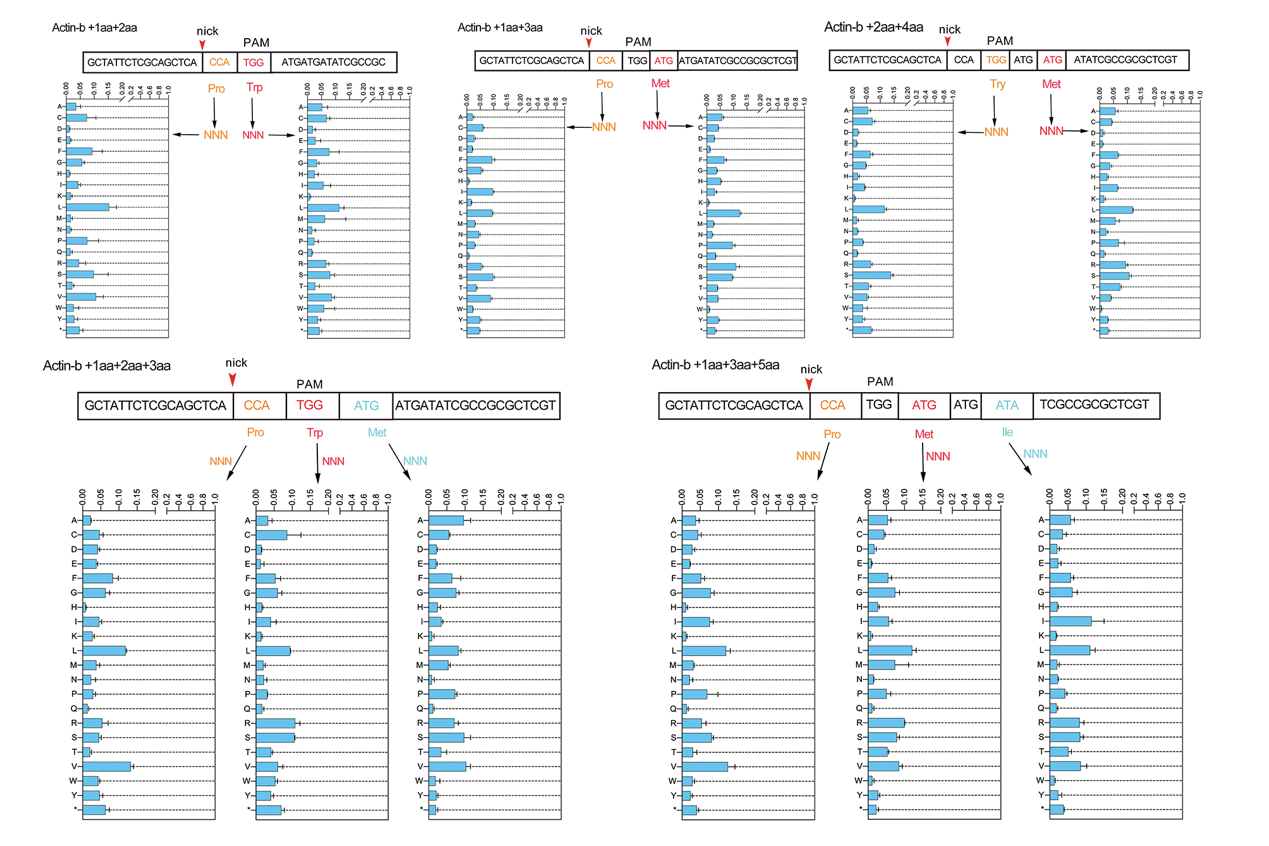


**Supplementary Figure 3.** Distribution of various codons in Actin-b gene edited by Random-PE.

Five types of amino acid substitutions, including +1aa+2aa, +1aa+3aa, +2aa+4aa, +1aa+2aa+3aa and +1aa+3aa+5aa, were established by Random-PE. The resulting substitutions were determined by HTS and corresponding codons were manually analyzed. Values and error bars reflect mean±s .d. of n=3 independent biological replicates.


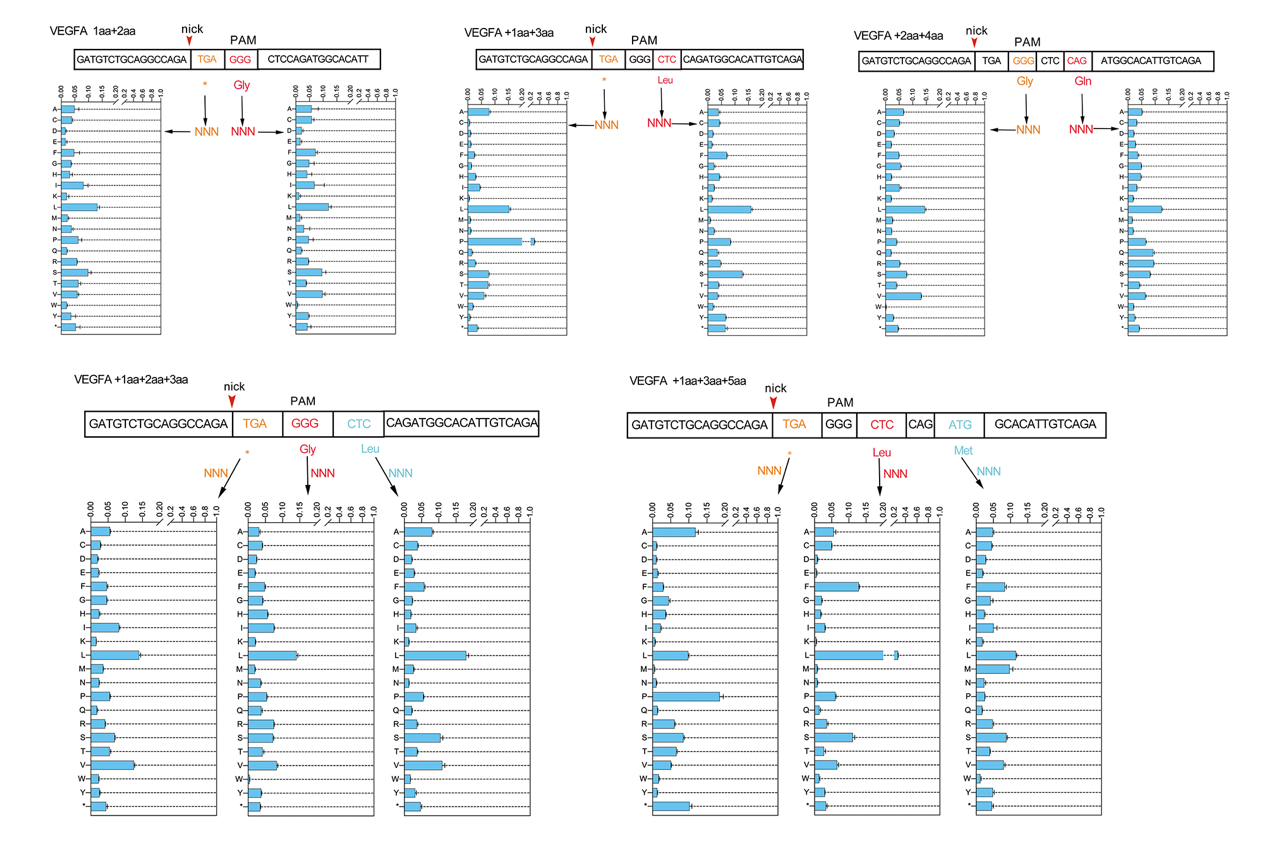


**Supplementary Figure 4.** Distribution of various codons in VEGFA gene edited by Random-PE.

Five types of amino acid substitutions, including +1aa+2aa, +1aa+3aa, +2aa+4aa, +1aa+2aa+3aa and +1aa+3aa+5aa, were established by Random-PE. The resulting substitutions were determined by HTS and corresponding codons were manually analyzed. Values and error bars reflect mean±s .d. of n=3 independent biological replicates.


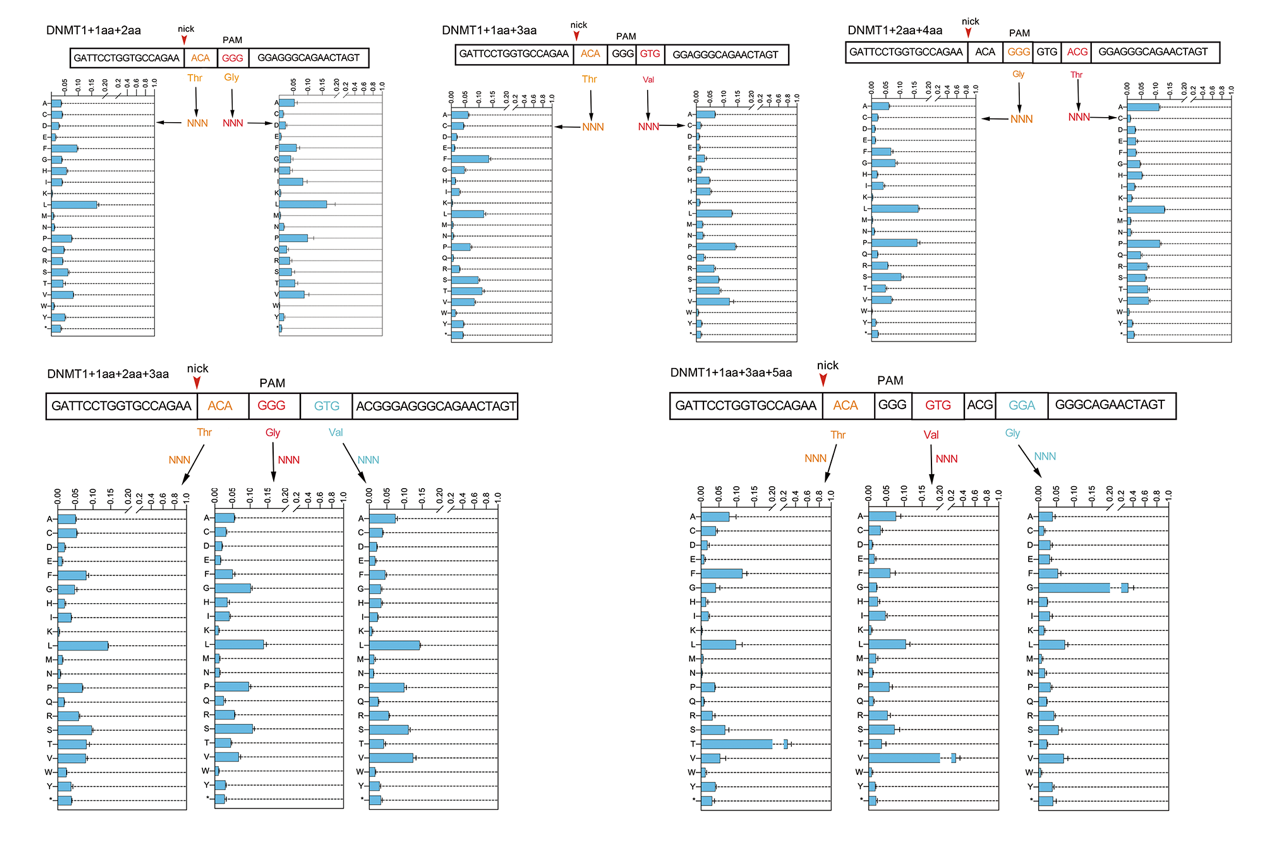


**Supplementary Figure 5.** Distribution of various codons in DNMT1 gene edited by Random-PE.

Five types of amino acid substitutions, including +1aa+2aa, +1aa+3aa, +2aa+4aa, +1aa+2aa+3aa and +1aa+3aa+5aa, were established by Random-PE. The resulting substitutions were determined by HTS and corresponding codons were manually analyzed. Values and error bars reflect mean±s .d. of n=3 independent biological replicates.

**Supplementary Table 1. List of the targets tested in this study.**

| sgRNA | Target sequence | Oligo-F | Oligo-R | Reference |
| --- | --- | --- | --- | --- |
| Actin-b | GCTATTCTCGCAGCTCACCA **TGG** | CACCGCTATTCTCGCAGCTCACCA | AAACTGGTGAGCTGCGAGAATAGC | This study |
| Actin-b +48 nick | GAAGCCGGCCTTGCACATGC **CGG** | CACCGAAGCCGGCCTTGCACATGC | AAACGCATGTGCAAGGCCGGCTTC | This study |
| VEGFA | GATGTCTGCAGGCCAGATGA **TGG** | CACCGATGTCTGCAGGCCAGATGA | AAACTCGCCCTGGGCTCTCTGTACATC | [1] |
| VEGFA+57 nick | ATGTACAGAGAGCCCAGGGC **TGG** | CACCGATGTACAGAGAGCCCAGGG | AAACGGAGCGGCTTCTGGTTTGTC | [1] |
| DNMT1 | GATTCCTGGTGCCAGAAACA **GGG** | CACCGATTCCTGGTGCCAGAAACA | AAACTGTTTCTGGCACCAGGAATC | [1] |
| DNMT1+49 nick | CCCTTCAGCTAAAATAAAGG **AGG** | CACCGCCCTTCAGCTAAAATAAAGG | AAACCCTTTATTTTAGCTGAAGGGC | [1] |

**Supplementary Table 2. Sequences of primers used for mammalian cell genomic DNA amplification.**

| Target site | chromosome | Forward primer | Reverse primer |
| --- | --- | --- | --- |
| Actin-b | Chr7 | CCCCCTGGCGGCCTA | CACGATGGAGGGGAAGACG |
| VEGFA | Chr6 | CTGACTAACCCCGGAACCAC | TTTGCTCCTGGACCCCCTAT |
| DNMT1 | Chr19 | AGTCCCGTGCAAATCACGAA | CCGTGAACGTTCCCTTAGCA |

**Supplementary Table 3. Sequences of pegRNAs used in mammalian cell experiments.**

| pegRNA | spacer | 3' extension (5' to 3') | PBS length  (nt) | HA  length (nt) |
| --- | --- | --- | --- | --- |
| DNMT1+6 G-C | GATTCCTGGTGCCAGAAACA | GTCACGCCTGTTTCTGGCACCAGG | 13 | 11 |
| Actin-b-KI-5N-  HA=10 | GCTATTCTCGCAGCTCACCA | TCATCCATGGNNNNNTGAGCTGCGAGAA | 13 | 10 |
| Actin-b-KI-8N-  HA=10 | GCTATTCTCGCAGCTCACCA | TCATCCATGGNNNNNNNNTGAGCTGCGAGAA | 13 | 10 |
| Actin-b-KI-10N-  HA=10 | GCTATTCTCGCAGCTCACCA | TCATCCATGGNNNNNNNNNNTGAGCTGCGAGAA | 13 | 10 |
| VEGFA-KI-5N-  HA=10 | GATGTCTGCAGGCCAGATGA | GGAGCCCTCANNNNNTCTGGCCTGCAGA | 13 | 10 |
| VEGFA-KI-8N-  HA=10 | GATGTCTGCAGGCCAGATGA | GGAGCCCTCANNNNNNNNTCTGGCCTGCAGA | 13 | 10 |
| VEGFA-KI-10N-  HA=10 | GATGTCTGCAGGCCAGATGA | GGAGCCCTCANNNNNNNNNNTCTGGCCTGCAGA | 13 | 10 |
| DNMT1-KI-5N-  HA=10 | GATTCCTGGTGCCAGAAACA | TCACCCCTGTNNNNNTTCTGGCACCAGG | 13 | 10 |
| DNMT1-KI-8N-  HA=10 | GATTCCTGGTGCCAGAAACA | TCACCCCTGTNNNNNNNNTTCTGGCACCAGG | 13 | 10 |
| DNMT1-KI-10N-  HA=10 | GATTCCTGGTGCCAGAAACA | TCACCCCTGTNNNNNNNNNNTTCTGGCACCAGG | 13 | 10 |
| Actin-b-KI-5N-  HA=20 | TGAGTCAGAGGGACCCTTTG | GGCGATATCATCATCCATGGNNNNNTGAGCTGCGAGAA | 13 | 20 |
| Actin-b-KI-5N-  HA=30 | GCTTGGGGCCAGAAGTGTCC | CGACGAGCGCGGCGATATCATCATCCATGGNNNNNTGAGCTGCGAGAA | 13 | 30 |
| Actin-b-KI-8N-  HA=20 | GGCCCAGACTGAGCACGTGA | GGCGATATCATCATCCATGGNNNNNNNNTGAGCTGCGAGAA | 13 | 20 |
| Actin-b-KI-8N-  HA=30 | GGCCCAGACTGAGCACGTGA | CGACGAGCGCGGCGATATCATCATCCATGGNNNNNNNNTGAGCTGCGAGAA | 13 | 30 |
| Actin-b-KI-10N-  HA=20 | TGAGTCAGAGGGACCCTTTG | GGCGATATCATCATCCATGGNNNNNNNNNNTGAGCTGCGAGAA | 13 | 20 |
| Actin-b-KI-10N-  HA=30 | GCTTGGGGCCAGAAGTGTCC | CGACGAGCGCGGCGATATCATCATCCATGGNNNNNNNNNNTGAGCTGCGAGAA | 13 | 30 |
| VEGFA-KI-5N-  HA=20 | GATGTCTGCAGGCCAGATGA | TGTGCCATCTGGAGCCCTCANNNNNTCTGGCCTGCAGA | 13 | 20 |
| VEGFA-KI-5N-  HA=30 | GATGTCTGCAGGCCAGATGA | CCTCTGACAATGTGCCATCTGGAGCCCTCANNNNNTCTGGCCTGCAGA | 13 | 30 |
| VEGFA-KI-8N-  HA=20 | GATGTCTGCAGGCCAGATGA | TGTGCCATCTGGAGCCCTCANNNNNNNNTCTGGCCTGCAGA | 13 | 20 |
| VEGFA-KI-8N-  HA=30 | GATGTCTGCAGGCCAGATGA | CCTCTGACAATGTGCCATCTGGAGCCCTCANNNNNNNNTCTGGCCTGCAGA | 13 | 30 |
| VEGFA-KI-10N-  HA=20 | GATGTCTGCAGGCCAGATGA | TGTGCCATCTGGAGCCCTCANNNNNNNNNNTCTGGCCTGCAGA | 13 | 20 |
| VEGFA-KI-10N-  HA=30 | GATGTCTGCAGGCCAGATGA | CCTCTGACAATGTGCCATCTGGAGCCCTCANNNNNNNNNNTCTGGCCTGCAGA | 13 | 30 |
| DNMT1-KI-5N-  HA=20 | GATTCCTGGTGCCAGAAACA | TGCCCTCCCGTCACCCCTGTNNNNNTTCTGGCACCAGG | 13 | 20 |
| DNMT1-KI-5N-  HA=30 | GATTCCTGGTGCCAGAAACA | GGACTAGTTCTGCCCTCCCGTCACCCCTGTNNNNNTTCTGGCACCAGG | 13 | 30 |
| DNMT1-KI-8N-  HA=20 | GATTCCTGGTGCCAGAAACA | TGCCCTCCCGTCACCCCTGTNNNNNNNNTTCTGGCACCAGG | 13 | 20 |
| DNMT1-KI-8N-  HA=30 | GATTCCTGGTGCCAGAAACA | GGACTAGTTCTGCCCTCCCGTCACCCCTGTNNNNNNNNTTCTGGCACCAGG | 13 | 30 |
| DNMT1-KI-10N-  HA=20 | GATTCCTGGTGCCAGAAACA | TGCCCTCCCGTCACCCCTGTNNNNNNNNNNTTCTGGCACCAGG | 13 | 20 |
| DNMT1-KI-10N-  HA=30 | GATTCCTGGTGCCAGAAACA | GGACTAGTTCTGCCCTCCCGTCACCCCTGTNNNNNNNNNNTTCTGGCACCAGG | 13 | 30 |
| Actin-b  +1aa+2aa | GCTATTCTCGCAGCTCACCA | TCGACGACGAGCGCGGCGATATCATCATNNNNNNTGAGCTGCGAGAA | 13 | 28 |
| Actin-b  +1aa+3aa | GCTATTCTCGCAGCTCACCA | TCGACGACGAGCGCGGCGATATCATNNNCCANNNTGAGCTGCGAGAA | 13 | 25 |
| Actin-b  +2aa+4aa | GCTATTCTCGCAGCTCACCA | TCGACGACGAGCGCGGCGATATNNNCATNNNTGGTGAGCTGCGAGAA | 13 | 22 |
| Actin-b  +1aa+2aa+3aa | GCTATTCTCGCAGCTCACCA | TCGACGACGAGCGCGGCGATATCATNNNNNNNNNTGAGCTGCGAGAA | 13 | 25 |
| Actin-b  +1aa+3aa+5aa | GCTATTCTCGCAGCTCACCA | TCGACGACGAGCGCGGCGANNNCATNNNCCANNNTGAGCTGCGAGAA | 13 | 19 |
| VEGFA  +1aa+2aa | GATGTCTGCAGGCCAGATGA | TGTCCCTCTGACAATGTGCCATCTGGAGNNNNNNTCTGGCCTGCAGA | 13 | 28 |
| VEGFA  +1aa+3aa | GATGTCTGCAGGCCAGATGA | TGTCCCTCTGACAATGTGCCATCTGNNNCCCNNNTCTGGCCTGCAGA | 13 | 25 |
| VEGFA  +2aa+4aa | GATGTCTGCAGGCCAGATGA | TGTCCCTCTGACAATGTGCCATNNNGAGNNNTCATCTGGCCTGCAGA | 13 | 22 |
| VEGFA  +1aa+2aa+3aa | GATGTCTGCAGGCCAGATGA | TGTCCCTCTGACAATGTGCCATCTGNNNNNNNNNTCTGGCCTGCAGA | 13 | 25 |
| VEGFA  +1aa+3aa+5aa | GATGTCTGCAGGCCAGATGA | TGTCCCTCTGACAATGTGCNNNCTGNNNCCCNNNTCTGGCCTGCAGA | 13 | 19 |
| DNMT1+  1aa+2aa | GATTCCTGGTGCCAGAAACA | GGACTAGTTCTGCCCTCCCGTCACNNNNNNTTCTGGCACCAGG | 13 | 24 |
| DNMT1  +1aa+3aa | GATTCCTGGTGCCAGAAACA | GGACTAGTTCTGCCCTCCCGTNNNCCCNNNTTCTGGCACCAGG | 13 | 21 |
| DNMT1  +2aa+4aa | GATTCCTGGTGCCAGAAACA | GGACTAGTTCTGCCCTCCNNNCACNNNTGTTTCTGGCACCAGG | 13 | 18 |
| DNMT1  +1aa+2aa+3aa | GATTCCTGGTGCCAGAAACA | GGACTAGTTCTGCCCTCCCGTNNNNNNNNNTTCTGGCACCAGG | 13 | 21 |
| DNMT1  +1aa+3aa+5aa | GATTCCTGGTGCCAGAAACA | GGACTAGTTCTGCCCNNNCGTNNNCCCNNNTTCTGGCACCAGG | 13 | 21 |

**Supplementary Table 4.** **Sequences of primers for amplification of pegRNAs.**

| pegRNA | Forward primer | Reverse primer |
| --- | --- | --- |
| DNMT1+6G-C | TCCGCGCACATTTCCCCGAA | R:AAAAAACCTGGTGCCAGAAACAGGCGTGACGCACCGACTCGGTGCCACTT |
| Actin-b-KI-5N | TCCGCGCACATTTCCCCGAA | R:AAAAAATTCTCGCAGCTCANNNNNCCATGGATGAGCACCGACTCGGTGCCACTT |
| Actin-b-KI-8N | TCCGCGCACATTTCCCCGAA | R:AAAAAATTCTCGCAGCTCANNNNNNNNCCATGGATGAGCACCGACTCGGTGCCACTT |
| Actin-b-KI-10N | TCCGCGCACATTTCCCCGAA | R:AAAAAATTCTCGCAGCTCANNNNNNNNNNCCATGGATGAGCACCGACTCGGTGCCACTT |
| VEGFA-KI-5N | TCCGCGCACATTTCCCCGAA | R:AAAAAATCTGCAGGCCAGANNNNNTGAGGGCTCCGCACCGACTCGGTGCCACTT |
| VEGFA-KI-8N | TCCGCGCACATTTCCCCGAA | R:AAAAAATCTGCAGGCCAGANNNNNNNNTGAGGGCTCCGCACCGACTCGGTGCCACTT |
| VEGFA-KI-10N | TCCGCGCACATTTCCCCGAA | R:AAAAAATCTGCAGGCCAGANNNNNNNNTGAGGGCTCCGCACCGACTCGGTGCCACTT |
| DNMT1-KI-5N | TCCGCGCACATTTCCCCGAA | R:AAAAAACCTGGTGCCAGAANNNNNACAGGGGTGAGCACCGACTCGGTGCCACTT |
| DNMT1-KI-8N | TCCGCGCACATTTCCCCGAA | R:AAAAAACCTGGTGCCAGAANNNNNNNNACAGGGGTGAGCACCGACTCGGTGCCACTT |
| DNMT1-KI-10N | TCCGCGCACATTTCCCCGAA | R:AAAAAACCTGGTGCCAGAANNNNNNNNNNACAGGGGTGAGCACCGACTCGGTGCCACTT |
| Actin-b-KI-5N-HA=10 | TCCGCGCACATTTCCCCGAA | R:AAAAAATTCTCGCAGCTCANNNNNCCATGGATGAGCACCGACTCGGTGCCACTT |
| Actin-b-KI-5N-HA=20 | TCCGCGCACATTTCCCCGAA | R1:CCATGGATGATGATATCGCCGCACCGACTCGGTGCCACTT  R2:AAAAAATTCTCGCAGCTCANNNNNCCATGGATGATGA |
| Actin-b-KI-5N-HA=30 | TCCGCGCACATTTCCCCGAA | R1:CCATGGATGATGATATCGCCGCGCTCGTCGGCACCGACTCGGTGCCACTT  R2:AAAAAATTCTCGCAGCTCANNNNNCCATGGATGATGA |
| Actin-b-KI-8N-HA=10 | TCCGCGCACATTTCCCCGAA | R:AAAAAACCTGGTGCCAGAANNNNNNNNACAGGGGTGAGCACCGACTCGGTGCCACTT |
| Actin-b-KI-8N-HA=20 | TCCGCGCACATTTCCCCGAA | R1:CCATGGATGATGATATCGCCGCACCGACTCGGTGCCACTT  R2:AAAAAATTCTCGCAGCTCANNNNNNNNCCATGGATGATGATATCGCC |
| Actin-b-KI-8N-HA=30 | TCCGCGCACATTTCCCCGAA | R1:CCATGGATGATGATATCGCCGCGCTCGTCGGCACCGACTCGGTGCCACTT  R2:AAAAAATTCTCGCAGCTCANNNNNNNNCCATGGATGATGATATCGCC |
| Actin-b-KI-10N-HA=10 | TCCGCGCACATTTCCCCGAA | R:AAAAAATTCTCGCAGCTCANNNNNNNNNNCCATGGATGAGCACCGACTCGGTGCCACTT |
| Actin-b-KI-10N-HA=20 | TCCGCGCACATTTCCCCGAA | R1:CCATGGATGATGATATCGCCGCACCGACTCGGTGCCACTT  R2:AAAAAATTCTCGCAGCTCANNNNNNNNNNCCATGGATGATGATATCGCCGCG |
| Actin-b-KI-10N-HA=30 | TCCGCGCACATTTCCCCGAA | R1:CCATGGATGATGATATCGCCGCGCTCGTCGGCACCGACTCGGTGCCACTT  R2:AAAAAATTCTCGCAGCTCANNNNNNNNNNCCATGGATGATGATATCGCCGCG |
| VEGFA-KI-5N-HA=10 | TCCGCGCACATTTCCCCGAA | R:AAAAAATCTGCAGGCCAGANNNNNTGAGGGCTCCGCACCGACTCGGTGCCACTT |
| VEGFA-KI-5N-HA=20 | TCCGCGCACATTTCCCCGAA | R1:TGAGGGCTCCAGATGGCACAGCACCGACTCGGTGCCACTT  R2:AAAAAATCTGCAGGCCAGANNNNNTGAGGGCTCCAGATGGCA |
| VEGFA-KI-5N-HA=30 | TCCGCGCACATTTCCCCGAA | R1:TGAGGGCTCCAGATGGCACATTGTCAGAGCACCGACTCGGTGCCACTT  R2:AAAAAATCTGCAGGCCAGANNNNNTGAGGGCTCCAGATGGCA |
| VEGFA-KI-8N-HA=10 | TCCGCGCACATTTCCCCGAA | R:AAAAAATCTGCAGGCCAGANNNNNNNNTGAGGGCTCCGCACCGACTCGGTGCCACTT |
| VEGFA-KI-8N-HA=20 | TCCGCGCACATTTCCCCGAA | R1:TGAGGGCTCCAGATGGCACAGCACCGACTCGGTGCCACTT  R2:AAAAAATCTGCAGGCCAGANNNNNNNNTGAGGGCTCCAGATGGCA |
| VEGFA-KI-8N-HA=30 | TCCGCGCACATTTCCCCGAA | R1:TGAGGGCTCCAGATGGCACATTGTCAGAGCACCGACTCGGTGCCACTT  R2:AAAAAATCTGCAGGCCAGANNNNNNNNTGAGGGCTCCAGATGGCA |
| VEGFA-KI-10N-HA=10 | TCCGCGCACATTTCCCCGAA | R:AAAAAATCTGCAGGCCAGANNNNNNNNTGAGGGCTCCGCACCGACTCGGTGCCACTT |
| VEGFA-KI-10N-HA=20 | TCCGCGCACATTTCCCCGAA | R1:TGAGGGCTCCAGATGGCACAGCACCGACTCGGTGCCACTT  R2:AAAAAATCTGCAGGCCAGANNNNNNNNNNTGAGGGCTCCAGATGGCA |
| VEGFA-KI-10N-HA=30 | TCCGCGCACATTTCCCCGAA | R1:TGAGGGCTCCAGATGGCACATTGTCAGAGCACCGACTCGGTGCCACTT  R2:AAAAAATCTGCAGGCCAGANNNNNNNNNNTGAGGGCTCCAGATGGCA |
| DNMT1-KI-5N-HA=10 | TCCGCGCACATTTCCCCGAA | R:AAAAAACCTGGTGCCAGAANNNNNACAGGGGTGAGCACCGACTCGGTGCCACTT |
| DNMT1-KI-5N-HA=20 | TCCGCGCACATTTCCCCGAA | R1:ACAGGGGTGACGGGAGGGCAGCACCGACTCGGTGCCACTT  R2:AAAAAACCTGGTGCCAGAANNNNNACAGGGGTGACGGGAGGGCA |
| DNMT1-KI-5N-HA=30 | TCCGCGCACATTTCCCCGAA | R1:ACAGGGGTGACGGGAGGGCAGAACTAGTCCGCACCGACTCGGTGCCACTT R2:AAAAAACCTGGTGCCAGAANNNNNACAGGGGTGACGGGAGGGCA |
| DNMT1-KI-8N-HA=10 | TCCGCGCACATTTCCCCGAA | R:AAAAAACCTGGTGCCAGAANNNNNNNNACAGGGGTGAGCACCGACTCGGTGCCACTT |
| DNMT1-KI-8N-HA=20 | TCCGCGCACATTTCCCCGAA | R1:ACAGGGGTGACGGGAGGGCAGCACCGACTCGGTGCCACTT  R2:AAAAAACCTGGTGCCAGAANNNNNNNNACAGGGGTGACGGGAGGGCA |
| DNMT1-KI-8N-HA=30 | TCCGCGCACATTTCCCCGAA | R1:ACAGGGGTGACGGGAGGGCAGAACTAGTCCGCACCGACTCGGTGCCACTT  R2:AAAAAACCTGGTGCCAGAANNNNNNNNACAGGGGTGACGGGAGGGCA |
| DNMT1-KI-10N-HA=10 | TCCGCGCACATTTCCCCGAA | R:AAAAAACCTGGTGCCAGAANNNNNNNNNNACAGGGGTGAGCACCGACTCGGTGCCACTT |
| DNMT1-KI-10N-HA=20 | TCCGCGCACATTTCCCCGAA | R1:ACAGGGGTGACGGGAGGGCAGCACCGACTCGGTGCCACTT  R2:AAAAAACCTGGTGCCAGAANNNNNNNNNNACAGGGGTGACGGGAGGGCA |
| DNMT1-KI-10N-HA=30 | TCCGCGCACATTTCCCCGAA | R1:ACAGGGGTGACGGGAGGGCAGAACTAGTCCGCACCGACTCGGTGCCACTT  R2:AAAAAACCTGGTGCCAGAANNNNNNNNNNACAGGGGTGACGGGAGGGCA |
| Actin-b +1aa+2aa | TCCGCGCACATTTCCCCGAA | R1:CCATGGATGATGATATCGCCGCGCTCGTCGTCGAGCACCGACTCGGTGCCACTT  R2:AAAAAATTCTCGCAGCTCANNNNNNATGATGATATCGCCGCGCT |
| Actin-b +1aa+3aa | TCCGCGCACATTTCCCCGAA | R1:CCATGGATGATGATATCGCCGCGCTCGTCGTCGAGCACCGACTCGGTGCCACTT  R2:AAAAAATTCTCGCAGCTCANNNTGGNNNATGATATCGCCGCGCT |
| Actin-b +2aa+4aa | TCCGCGCACATTTCCCCGAA | R1:CCATGGATGATGATATCGCCGCGCTCGTCGTCGAGCACCGACTCGGTGCCACTT  R2:AAAAAATTCTCGCAGCTCACCANNNATGNNNATATCGCCGCGCTCGTCGT |
| Actin-b +1aa+2aa+3aa | TCCGCGCACATTTCCCCGAA | R1:CCATGGATGATGATATCGCCGCGCTCGTCGTCGAGCACCGACTCGGTGCCACTT  R2:AAAAAATTCTCGCAGCTCANNNNNNNNNATGATATCGCCGCGCT |
| Actin-b +1aa+3aa+5aa | TCCGCGCACATTTCCCCGAA | R1:CCATGGATGATGATATCGCCGCGCTCGTCGTCGAGCACCGACTCGGTGCCACTT  R2:AAAAAATTCTCGCAGCTCANNNTGGNNNATGNNNTCGCCGCGCTCGTCGT |
| VEGFA +1aa+2aa | TCCGCGCACATTTCCCCGAA | R1:TGAGGGCTCCAGATGGCACATTGTCAGAGGGACAGCACCGACTCGGTGCCACTT  R2:AAAAAATCTGCAGGCCAGANNNNNNCTCCAGATGGCACATTGTCAGAG |
| VEGFA +1aa+3aa | TCCGCGCACATTTCCCCGAA | R1:TGAGGGCTCCAGATGGCACATTGTCAGAGGGACAGCACCGACTCGGTGCCACTT  R2:AAAAAATCTGCAGGCCAGANNNGGGNNNCAGATGGCACATTGTCAGAG |
| VEGFA +2aa+4aa | TCCGCGCACATTTCCCCGAA | R1:TGAGGGCTCCAGATGGCACATTGTCAGAGGGACAGCACCGACTCGGTGCCACTT  R2:AAAAAATCTGCAGGCCAGATGANNNCTCNNNATGGCACATTGTCAGAGGGACA |
| VEGFA +1aa+2aa+3 aa | TCCGCGCACATTTCCCCGAA | R1:TGAGGGCTCCAGATGGCACATTGTCAGAGGGACAGCACCGACTCGGTGCCACTT  R2:AAAAAATCTGCAGGCCAGANNNNNNNNNCAGATGGCACATTGTCAGAG |
| VEGFA  +1 aa+3aa+5aa | TCCGCGCACATTTCCCCGAA | R1:TGAGGGCTCCAGATGGCACATTGTCAGAGGGACAGCACCGACTCGGTGCCACTT  R2:AAAAAATCTGCAGGCCAGANNNGGGNNNCAGNNNGCACATTGTCAGAGGGACA |
| DNMT1  +1aa +2aa | TCCGCGCACATTTCCCCGAA | R1:ACAGGGGTGACGGGAGGGCAGAACTAGTCCGCACCGACTCGGTGCCACTT  R2:AAAAAACCTGGTGCCAGAANNNNNNGTGACGGGAGGGCAGAACTA |
| DNMT1  +1 aa +3 aa | TCCGCGCACATTTCCCCGAA | R1:ACAGGGGTGACGGGAGGGCAGAACTAGTCCGCACCGACTCGGTGCCACTT  R2:AAAAAACCTGGTGCCAGAANNNGGGNNNACGGGAGGGCAGAACTAGTC |
| DNMT1  +2 aa +4 aa | TCCGCGCACATTTCCCCGAA | R1:ACAGGGGTGACGGGAGGGCAGAACTAGTCCGCACCGACTCGGTGCCACTT  R2:AAAAAACCTGGTGCCAGAAACANNNGTGNNNGGAGGGCAGAACTAGTCC |
| DNMT1  +1aa +2aa+3aa | TCCGCGCACATTTCCCCGAA | R1:ACAGGGGTGACGGGAGGGCAGAACTAGTCCGCACCGACTCGGTGCCACTT  R2:AAAAAACCTGGTGCCAGAANNNNNNNNNACGGGAGGGCAGAACTAGTCC |
| DNMT1  +1aa+3aa+5 aa | TCCGCGCACATTTCCCCGAA | R1:ACAGGGGTGACGGGAGGGCAGAACTAGTCCGCACCGACTCGGTGCCACTT  R2:AAAAAACCTGGTGCCAGAANNNGGGNNNACGNNNGGGCAGAACTAGTCC |

**Supplementary Table 5.** **Sequences of primers used for HTS.**

| sample name | Primer Name | Sequence |
| --- | --- | --- |
| Actin-b-KI-5N | Actin-b -HTS -1-few | gtctagtCCCCCTGGCGGCCTA |
|  | Actin-b -HTS -rev | CACGATGGAGGGGAAGACG |
| Actin-b-KI-5N | Actin-b -HTS -2-few | gtcgtgaCCCCCTGGCGGCCTA |
|  | Actin-b -HTS -rev | CACGATGGAGGGGAAGACG |
| Actin-b-KI-5N | Actin-b -HTS -3-few | ctgatgtCCCCCTGGCGGCCTA |
|  | Actin-b -HTS -rev | CACGATGGAGGGGAAGACG |
| Actin-b-KI-8N | Actin-b -HTS -4-few | ctgtagaCCCCCTGGCGGCCTA |
|  | Actin-b -HTS -rev | CACGATGGAGGGGAAGACG |
| Actin-b-KI-8N | Actin-b -HTS -5-few | gaacactCCCCCTGGCGGCCTA |
|  | Actin-b -HTS -rev | CACGATGGAGGGGAAGACG |
| Actin-b-KI-8N | Actin-b -HTS -6-few | gatctcaCCCCCTGGCGGCCTA |
|  | Actin-b -HTS -rev | CACGATGGAGGGGAAGACG |
| Actin-b-KI-10N | Actin-b -HTS -7-few | gacatctCCCCCTGGCGGCCTA |
|  | Actin-b -HTS -rev | CACGATGGAGGGGAAGACG |
| Actin-b-KI-10N | Actin-b -HTS -8-few | gtctagtCCCCCTGGCGGCCTA |
|  | Actin-b -HTS -rev | CACGATGGAGGGGAAGACG |
| VEGFA-KI-5N | VEGFA -HTS -1-few | ctctcagCCAAAGGACCCCAGTCACTC |
|  | VEGFA -HTS -rev | TGGGACTGGAGTTGCTTCAT |
| VEGFA-KI-5N | VEGFA -HTS -2-few | ctctgtaCCAAAGGACCCCAGTCACTC |
|  | VEGFA -HTS -rev | TGGGACTGGAGTTGCTTCAT |
| VEGFA-KI-5N | VEGFA -HTS -3-few | ctgatgtCCAAAGGACCCCAGTCACTC |
|  | VEGFA -HTS -rev | TGGGACTGGAGTTGCTTCAT |
| VEGFA-KI-8N | VEGFA -HTS -4-few | caagtagCCAAAGGACCCCAGTCACTC |
|  | VEGFA -HTS -rev | TGGGACTGGAGTTGCTTCAT |
| VEGFA-KI-8N | VEGFA -HTS -5-few | caagcttCCAAAGGACCCCAGTCACTC |
|  | VEGFA -HTS -rev | TGGGACTGGAGTTGCTTCAT |
| VEGFA-KI-8N | VEGFA -HTS -6-few | catcttgCCAAAGGACCCCAGTCACTC |
|  | VEGFA -HTS -rev | TGGGACTGGAGTTGCTTCAT |
| VEGFA-KI-10N | VEGFA -HTS -7-few | catcgacCCAAAGGACCCCAGTCACTC |
|  | VEGFA -HTS -rev | TGGGACTGGAGTTGCTTCAT |
| VEGFA-KI-10N | VEGFA -HTS -8-few | catgatcCCAAAGGACCCCAGTCACTC |
|  | VEGFA -HTS -rev | TGGGACTGGAGTTGCTTCAT |
| VEGFA-KI-10N | VEGFA -HTS -9-few | catgcaaCCAAAGGACCCCAGTCACTC |
|  | VEGFA -HTS -rev | TGGGACTGGAGTTGCTTCAT |
| DNMT1-KI-5N | DNMT1 -HTS -17-few | ctcgtatACCACACATGTGAACGGACA |
|  | DNMT1 -HTS -rev | CGTGTTCCCCAGAGTGACTT |
| DNMT1-KI-5N | DNMT1 -HTS -17-few | ctcgtatACCACACATGTGAACGGACA |
|  | DNMT1 -HTS -rev | CGTGTTCCCCAGAGTGACTT |
| DNMT1-KI-5N | DNMT1 -HTS -24-few | attgctcACCACACATGTGAACGGACA |
|  | DNMT1 -HTS -rev | CGTGTTCCCCAGAGTGACTT |
| DNMT1-KI-8N | DNMT1 -HTS -18-few | ctgctaaACCACACATGTGAACGGACA |
|  | DNMT1 -HTS -rev | CGTGTTCCCCAGAGTGACTT |
| DNMT1-KI-8N | DNMT1 -HTS -18-few | ctgctaaACCACACATGTGAACGGACA |
|  | DNMT1 -HTS -rev | CGTGTTCCCCAGAGTGACTT |
| DNMT1-KI-8N | DNMT1 -HTS -25-few | atcaagcACCACACATGTGAACGGACA |
|  | DNMT1 -HTS -rev | CGTGTTCCCCAGAGTGACTT |
| DNMT1-KI-10N | DNMT1 -HTS -19-few | ctggatgACCACACATGTGAACGGACA |
|  | DNMT1 -HTS -rev | CGTGTTCCCCAGAGTGACTT |
| DNMT1-KI-10N | DNMT1 -HTS -19-few | ctggatgACCACACATGTGAACGGACA |
|  | DNMT1 -HTS -rev | CGTGTTCCCCAGAGTGACTT |
| DNMT1-KI-10N | DNMT1 -HTS -26-few | atccggaaCCACACATGTGAACGGACA |
|  | DNMT1 -HTS -rev | CGTGTTCCCCAGAGTGACTT |
| Actin-b-KI-5N-HA=10 | Actin-b -HTS -10-few | ctgatgtCCCCCTGGCGGCCTA |
|  | Actin-b -HTS -rev | CACGATGGAGGGGAAGACG |
| Actin-b-KI-5N-HA=10 | Actin-b -HTS -10-few | ctgatgtCCCCCTGGCGGCCTA |
|  | Actin-b -HTS -rev | CACGATGGAGGGGAAGACG |
| Actin-b-KI-5N-HA=10 | Actin-b -HTS -16-few | gactacaCCCCCTGGCGGCCTA |
|  | Actin-b -HTS -rev | CACGATGGAGGGGAAGACG |
| Actin-b-KI-5N-HA=20 | Actin-b -HTS -11-few | ctgtagaCCCCCTGGCGGCCTA |
|  | Actin-b -HTS -rev | CACGATGGAGGGGAAGACG |
| Actin-b-KI-5N-HA=20 | Actin-b -HTS -11-few | ctgtagaCCCCCTGGCGGCCTA |
|  | Actin-b -HTS -rev | CACGATGGAGGGGAAGACG |
| Actin-b-KI-5N-HA=20 | Actin-b -HTS -17-few | gactcacCCCCCTGGCGGCCTA |
|  | Actin-b -HTS -rev | CACGATGGAGGGGAAGACG |
| Actin-b-KI-5N-HA=30 | Actin-b -HTS -12-few | gaacactCCCCCTGGCGGCCTA |
|  | Actin-b -HTS -rev | CACGATGGAGGGGAAGACG |
| Actin-b-KI-5N-HA=30 | Actin-b -HTS -12-few | gaacactCCCCCTGGCGGCCTA |
|  | Actin-b -HTS -rev | CACGATGGAGGGGAAGACG |
| Actin-b-KI-5N-HA=30 | Actin-b -HTS -12-few | gaacactCCCCCTGGCGGCCTA |
|  | Actin-b -HTS -rev | CACGATGGAGGGGAAGACG |
| Actin-b-KI-8N-HA=10 | Actin-b -HTS -13-few | gatctcaCCCCCTGGCGGCCTA |
|  | Actin-b -HTS -rev | CACGATGGAGGGGAAGACG |
| Actin-b-KI-8N-HA=10 | Actin-b -HTS -13-few | gatctcaCCCCCTGGCGGCCTA |
|  | Actin-b -HTS -rev | CACGATGGAGGGGAAGACG |
| Actin-b-KI-8N-HA=10 | Actin-b -HTS -14-few | gacatctCCCCCTGGCGGCCTA |
|  | Actin-b -HTS -rev | CACGATGGAGGGGAAGACG |
| Actin-b-KI-8N-HA=20 | Actin-b -HTS -14-few | gacatctCCCCCTGGCGGCCTA |
|  | Actin-b -HTS -rev | CACGATGGAGGGGAAGACG |
| Actin-b-KI-8N-HA=20 | Actin-b -HTS -14-few | gacatctCCCCCTGGCGGCCTA |
|  | Actin-b -HTS -rev | CACGATGGAGGGGAAGACG |
| Actin-b-KI-8N-HA=20 | Actin-b -HTS -15-few | gacagtaCCCCCTGGCGGCCTA |
|  | Actin-b -HTS -rev | CACGATGGAGGGGAAGACG |
| Actin-b-KI-8N-HA=30 | Actin-b -HTS -15-few | gacagtaCCCCCTGGCGGCCTA |
|  | Actin-b -HTS -rev | CACGATGGAGGGGAAGACG |
| Actin-b-KI-8N-HA=30 | Actin-b -HTS -15-few | gacagtaCCCCCTGGCGGCCTA |
|  | Actin-b -HTS -rev | CACGATGGAGGGGAAGACG |
| Actin-b-KI-8N-HA=30 | Actin-b -HTS -15-few | gacagtaCCCCCTGGCGGCCTA |
|  | Actin-b -HTS -rev | CACGATGGAGGGGAAGACG |
| Actin-b-KI-10N-HA=10 | Actin-b -HTS -1-few | gtctagtCCCCCTGGCGGCCTA |
|  | Actin-b -HTS -rev | CACGATGGAGGGGAAGACG |
| Actin-b-KI-10N-HA=10 | Actin-b -HTS -2-few | gtcgtgaCCCCCTGGCGGCCTA |
|  | Actin-b -HTS -rev | CACGATGGAGGGGAAGACG |
| Actin-b-KI-10N-HA=10 | Actin-b -HTS -3-few | ctgatgtCCCCCTGGCGGCCTA |
|  | Actin-b -HTS -rev | CACGATGGAGGGGAAGACG |
| Actin-b-KI-10N-HA=20 | Actin-b -HTS -4-few | ctgtagaCCCCCTGGCGGCCTA |
|  | Actin-b -HTS -rev | CACGATGGAGGGGAAGACG |
| Actin-b-KI-10N-HA=20 | Actin-b -HTS -5-few | gaacactCCCCCTGGCGGCCTA |
|  | Actin-b -HTS -rev | CACGATGGAGGGGAAGACG |
| Actin-b-KI-10N-HA=20 | Actin-b -HTS -6-few | gatctcaCCCCCTGGCGGCCTA |
|  | Actin-b -HTS -rev | CACGATGGAGGGGAAGACG |
| Actin-b-KI-10N-HA=30 | Actin-b -HTS -7-few | gacatctCCCCCTGGCGGCCTA |
|  | Actin-b -HTS -rev | CACGATGGAGGGGAAGACG |
| Actin-b-KI-10N-HA=30 | Actin-b -HTS -8-few | gtctagtCCCCCTGGCGGCCTA |
|  | Actin-b -HTS -rev | CACGATGGAGGGGAAGACG |
| Actin-b-KI-10N-HA=30 | Actin-b -HTS -9-few | gactacaCCCCCTGGCGGCCTA |
|  | Actin-b -HTS -rev | CACGATGGAGGGGAAGACG |
| VEGFA-KI-5N-HA=10 | VEGFA -HTS -11-few | cacactgCCAAAGGACCCCAGTCACTC |
|  | VEGFA -HTS -rev | TGGGACTGGAGTTGCTTCAT |
| VEGFA-KI-5N-HA=10 | VEGFA -HTS -18-few | cagtctaCCAAAGGACCCCAGTCACTC |
|  | VEGFA -HTS -rev | TGGGACTGGAGTTGCTTCAT |
| VEGFA-KI-5N-HA=10 | VEGFA -HTS -19-few | ctagacaCCAAAGGACCCCAGTCACTC |
|  | VEGFA -HTS -rev | TGGGACTGGAGTTGCTTCAT |
| VEGFA-KI-5N-HA=20 | VEGFA -HTS -11-few | cacactgCCAAAGGACCCCAGTCACTC |
|  | VEGFA -HTS -rev | TGGGACTGGAGTTGCTTCAT |
| VEGFA-KI-5N-HA=20 | VEGFA -HTS -11-few | cacactgCCAAAGGACCCCAGTCACTC |
|  | VEGFA -HTS -rev | TGGGACTGGAGTTGCTTCAT |
| VEGFA-KI-5N-HA=20 | VEGFA -HTS -12-few | cacttgaCCAAAGGACCCCAGTCACTC |
|  | VEGFA -HTS -rev | TGGGACTGGAGTTGCTTCAT |
| VEGFA-KI-5N-HA=30 | VEGFA -HTS -12-few | cacttgaCCAAAGGACCCCAGTCACTC |
|  | VEGFA -HTS -rev | TGGGACTGGAGTTGCTTCAT |
| VEGFA-KI-5N-HA=30 | VEGFA -HTS -12-few | cacttgaCCAAAGGACCCCAGTCACTC |
|  | VEGFA -HTS -rev | TGGGACTGGAGTTGCTTCAT |
| VEGFA-KI-5N-HA=30 | VEGFA -HTS -12-few | cacttgaCCAAAGGACCCCAGTCACTC |
|  | VEGFA -HTS -rev | TTGTAGAAGGTGTGGTGCCA |
| VEGFA-KI-8N-HA=10 | VEGFA -HTS -14-few | cagatcaCCAAAGGACCCCAGTCACTC |
|  | VEGFA -HTS -rev | TGGGACTGGAGTTGCTTCAT |
| VEGFA-KI-8N-HA=10 | VEGFA -HTS -13-few | cactgatCCAAAGGACCCCAGTCACTC |
|  | VEGFA -HTS -rev | TGGGACTGGAGTTGCTTCAT |
| VEGFA-KI-8N-HA=10 | VEGFA -HTS -13-few | cactgatCCAAAGGACCCCAGTCACTC |
|  | VEGFA -HTS -rev | TGGGACTGGAGTTGCTTCAT |
| VEGFA-KI-8N-HA=20 | VEGFA -HTS -11-few | cacactgCCAAAGGACCCCAGTCACTC |
|  | VEGFA -HTS -rev | TGGGACTGGAGTTGCTTCAT |
| VEGFA-KI-8N-HA=20 | VEGFA -HTS -14-few | cagatcaCCAAAGGACCCCAGTCACTC |
|  | VEGFA -HTS -rev | TGGGACTGGAGTTGCTTCAT |
| VEGFA-KI-8N-HA=20 | VEGFA -HTS -14-few | cagatcaCCAAAGGACCCCAGTCACTC |
|  | VEGFA -HTS -rev | TGGGACTGGAGTTGCTTCAT |
| VEGFA-KI-8N-HA=30 | VEGFA -HTS -15-few | cagacatCCAAAGGACCCCAGTCACTC |
|  | VEGFA -HTS -rev | TGGGACTGGAGTTGCTTCAT |
| VEGFA-KI-8N-HA=30 | VEGFA -HTS -15-few | cagacatCCAAAGGACCCCAGTCACTC |
|  | VEGFA -HTS -rev | TGGGACTGGAGTTGCTTCAT |
| VEGFA-KI-8N-HA=30 | VEGFA -HTS -12-few | cacttgaCCAAAGGACCCCAGTCACTC |
|  | VEGFA -HTS -rev | TGGGACTGGAGTTGCTTCAT |
| VEGFA-KI-10N-HA=10 | VEGFA -HTS -1-few | ctctcagCCAAAGGACCCCAGTCACTC |
|  | VEGFA -HTS -rev | TGGGACTGGAGTTGCTTCAT |
| VEGFA-KI-10N-HA=10 | VEGFA -HTS -2-few | ctctgtaCCAAAGGACCCCAGTCACTC |
|  | VEGFA -HTS -rev | TGGGACTGGAGTTGCTTCAT |
| VEGFA-KI-10N-HA=10 | VEGFA -HTS -3-few | ctgatgtCCAAAGGACCCCAGTCACTC |
|  | VEGFA -HTS -rev | TGGGACTGGAGTTGCTTCAT |
| VEGFA-KI-10N-HA=20 | VEGFA -HTS -4-few | caagtagCCAAAGGACCCCAGTCACTC |
|  | VEGFA -HTS -rev | TGGGACTGGAGTTGCTTCAT |
| VEGFA-KI-10N-HA=20 | VEGFA -HTS -5-few | caagcttCCAAAGGACCCCAGTCACTC |
|  | VEGFA -HTS -rev | TGGGACTGGAGTTGCTTCAT |
| VEGFA-KI-10N-HA=20 | VEGFA -HTS -6-few | catcttgCCAAAGGACCCCAGTCACTC |
|  | VEGFA -HTS -rev | TGGGACTGGAGTTGCTTCAT |
| VEGFA-KI-10N-HA=30 | VEGFA -HTS -7-few | catcgacCCAAAGGACCCCAGTCACTC |
|  | VEGFA -HTS -rev | TGGGACTGGAGTTGCTTCAT |
| VEGFA-KI-10N-HA=30 | VEGFA -HTS -8-few | catgatcCCAAAGGACCCCAGTCACTC |
|  | VEGFA -HTS -rev | TGGGACTGGAGTTGCTTCAT |
| VEGFA-KI-10N-HA=30 | VEGFA -HTS -9-few | catgcaaCCAAAGGACCCCAGTCACTC |
|  | VEGFA -HTS -rev | TGGGACTGGAGTTGCTTCAT |
| DNMT1-KI-5N-HA=10 | DNMT1 -HTS -1-few | gtgaacaaCCACACATGTGAACGGACA |
|  | DNMT1 -HTS -rev | CGTGTTCCCCAGAGTGACTT |
| DNMT1-KI-5N-HA=10 | DNMT1 -HTS -20-few | ccaatgaaCCACACATGTGAACGGACA |
|  | DNMT1 -HTS -rev | CGTGTTCCCCAGAGTGACTT |
| DNMT1-KI-5N-HA=10 | DNMT1 -HTS -1-few | gtgaacaaCCACACATGTGAACGGACA |
|  | DNMT1 -HTS -rev | CGTGTTCCCCAGAGTGACTT |
| DNMT1-KI-5N-HA=20 | DNMT1 -HTS -2-few | gtgactcACCACACATGTGAACGGACA |
|  | DNMT1 -HTS -rev | CGTGTTCCCCAGAGTGACTT |
| DNMT1-KI-5N-HA=20 | DNMT1 -HTS -2-few | gtgactcACCACACATGTGAACGGACA |
|  | DNMT1 -HTS -rev | CGTGTTCCCCAGAGTGACTT |
| DNMT1-KI-5N-HA=20 | DNMT1 -HTS -21-few | aagatgcACCACACATGTGAACGGACA |
|  | DNMT1 -HTS -rev | CGTGTTCCCCAGAGTGACTT |
| DNMT1-KI-5N-HA=30 | DNMT1 -HTS -3-few | gtgtcaaACCACACATGTGAACGGACA |
|  | DNMT1 -HTS -rev | CGTGTTCCCCAGAGTGACTT |
| DNMT1-KI-5N-HA=30 | DNMT1 -HTS -3-few | gtgtcaaACCACACATGTGAACGGACA |
|  | DNMT1 -HTS -rev | CGTGTTCCCCAGAGTGACTT |
| DNMT1-KI-5N-HA=30 | DNMT1 -HTS -22-few | aagcgtgACCACACATGTGAACGGACA |
|  | DNMT1 -HTS -rev | CGTGTTCCCCAGAGTGACTT |
| DNMT1-KI-8N-HA=10 | DNMT1 -HTS -4-few | aactgtcACCACACATGTGAACGGACA |
|  | DNMT1 -HTS -rev | CGTGTTCCCCAGAGTGACTT |
| DNMT1-KI-8N-HA=10 | DNMT1 -HTS -4-few | aactgtcACCACACATGTGAACGGACA |
|  | DNMT1 -HTS -rev | CGTGTTCCCCAGAGTGACTT |
| DNMT1-KI-8N-HA=10 | DNMT1 -HTS -23-few | attctcgACCACACATGTGAACGGACA |
|  | DNMT1 -HTS -rev | CGTGTTCCCCAGAGTGACTT |
| DNMT1-KI-8N-HA=20 | DNMT1 -HTS -5-few | aacgtgtACCACACATGTGAACGGACA |
|  | DNMT1 -HTS -rev | CGTGTTCCCCAGAGTGACTT |
| DNMT1-KI-8N-HA=20 | DNMT1 -HTS -5-few | aacgtgtACCACACATGTGAACGGACA |
|  | DNMT1 -HTS -rev | CGTGTTCCCCAGAGTGACTT |
| DNMT1-KI-8N-HA=20 | DNMT1 -HTS -24-few | attgctcACCACACATGTGAACGGACA |
|  | DNMT1 -HTS -rev | CGTGTTCCCCAGAGTGACTT |
| DNMT1-KI-8N-HA=30 | DNMT1 -HTS -6-few | aagagctACCACACATGTGAACGGACA |
|  | DNMT1 -HTS -rev | CGTGTTCCCCAGAGTGACTT |
| DNMT1-KI-8N-HA=30 | DNMT1 -HTS -6-few | aagagctACCACACATGTGAACGGACA |
|  | DNMT1 -HTS -rev | CGTGTTCCCCAGAGTGACTT |
| DNMT1-KI-8N-HA=30 | DNMT1 -HTS -25-few | atcaagcACCACACATGTGAACGGACA |
|  | DNMT1 -HTS -rev | CGTGTTCCCCAGAGTGACTT |
| DNMT1-KI-10N-HA=10 | DNMT1 -HTS -7-few | aagtcagACCACACATGTGAACGGACA |
|  | DNMT1 -HTS -rev | CGTGTTCCCCAGAGTGACTT |
| DNMT1-KI-10N-HA=10 | DNMT1 -HTS -7-few | aagtcagACCACACATGTGAACGGACA |
|  | DNMT1 -HTS -rev | CGTGTTCCCCAGAGTGACTT |
| DNMT1-KI-10N-HA=10 | DNMT1 -HTS -26-few | atccggaaCCACACATGTGAACGGACA |
|  | DNMT1 -HTS -rev | CGTGTTCCCCAGAGTGACTT |
| DNMT1-KI-10N-HA=20 | DNMT1 -HTS -8-few | atcagtgACCACACATGTGAACGGACA |
|  | DNMT1 -HTS -rev | CGTGTTCCCCAGAGTGACTT |
| DNMT1-KI-10N-HA=20 | DNMT1 -HTS -8-few | atcagtgACCACACATGTGAACGGACA |
|  | DNMT1 -HTS -rev | CGTGTTCCCCAGAGTGACTT |
| DNMT1-KI-10N-HA=20 | DNMT1 -HTS -27-few | atcgaagACCACACATGTGAACGGACA |
|  | DNMT1 -HTS -rev | CGTGTTCCCCAGAGTGACTT |
| DNMT1-KI-10N-HA=30 | DNMT1 -HTS -9-few | atctgctACCACACATGTGAACGGACA |
|  | DNMT1 -HTS -rev | CGTGTTCCCCAGAGTGACTT |
| DNMT1-KI-10N-HA=30 | DNMT1 -HTS -9-few | atctgctACCACACATGTGAACGGACA |
|  | DNMT1 -HTS -rev | CGTGTTCCCCAGAGTGACTT |
| DNMT1-KI-10N-HA=30 | DNMT1 -HTS -28-few | atcgtccACCACACATGTGAACGGACA |
|  | DNMT1 -HTS -rev | CGTGTTCCCCAGAGTGACTT |
| Actin-b+1aa+2aa | Actin-b -HTS -16-few | gactacaCCCCCTGGCGGCCTA |
|  | Actin-b -HTS -rev | CACGATGGAGGGGAAGACG |
| Actin-b+1aa+2aa | Actin-b -HTS -16-few | gactacaCCCCCTGGCGGCCTA |
|  | Actin-b -HTS -rev | CACGATGGAGGGGAAGACG |
| Actin-b+1aa+2aa | Actin-b -HTS -17-few | gactcacCCCCCTGGCGGCCTA |
|  | Actin-b -HTS -rev | CACGATGGAGGGGAAGACG |
| Actin-b+1aa+3aa | Actin-b -HTS -17-few | gactcacCCCCCTGGCGGCCTA |
|  | Actin-b -HTS -rev | CACGATGGAGGGGAAGACG |
| Actin-b+1aa+3aa | Actin-b -HTS -17-few | gactcacCCCCCTGGCGGCCTA |
|  | Actin-b -HTS -rev | CACGATGGAGGGGAAGACG |
| Actin-b+1aa+3aa | Actin-b -HTS -18few | gagtcgtCCCCCTGGCGGCCTA |
|  | Actin-b -HTS -rev | CACGATGGAGGGGAAGACG |
| Actin-b+2aa+4aa | Actin-b -HTS -20few | gtagtctCCCCCTGGCGGCCTA |
|  | Actin-b -HTS -rev | CACGATGGAGGGGAAGACG |
| Actin-b+2aa+4aa | Actin-b -HTS -20few | gtagtctCCCCCTGGCGGCCTA |
|  | Actin-b -HTS -rev | CACGATGGAGGGGAAGACG |
| Actin-b+2aa+4aa | Actin-b -HTS -10-few | ctgatgtCCCCCTGGCGGCCTA |
|  | Actin-b -HTS -rev | CACGATGGAGGGGAAGACG |
| Actin-b+1aa+2aa+3aa | Actin-b -HTS -21-few | gtcacatCCCCCTGGCGGCCTA |
|  | Actin-b -HTS -rev | CACGATGGAGGGGAAGACG |
| Actin-b+1aa+2aa+3aa | Actin-b -HTS -21-few | gtcacatCCCCCTGGCGGCCTA |
|  | Actin-b -HTS -rev | CACGATGGAGGGGAAGACG |
| Actin-b+1aa+2aa+3aa | Actin-b -HTS -2-few | gtcgtgaCCCCCTGGCGGCCTA |
|  | Actin-b -HTS -rev | CACGATGGAGGGGAAGACG |
| Actin-b+1aa+3aa+5aa | Actin-b -HTS -19-few | gtacagaCCCCCTGGCGGCCTA |
|  | Actin-b -HTS -rev | TGGGACTGGAGTTGCTTCAT |
| Actin-b+1aa+3aa+5aa | Actin-b -HTS -19-few | gtacagaCCCCCTGGCGGCCTA |
|  | Actin-b -HTS -rev | CACGATGGAGGGGAAGACG |
| Actin-b+1aa+3aa+5aa | Actin-b -HTS -8-few | ctcgtatCCCCCTGGCGGCCTA |
|  | Actin-b -HTS -rev | TGGGACTGGAGTTGCTTCAT |
| Untreated- Actin-b | Actin-b -HTS -22-few | ccaatgaCCCCCTGGCGGCCTA |
|  | Actin-b -HTS -rev | TGGGACTGGAGTTGCTTCAT |
| Untreated- Actin-b | Actin-b -HTS -22-few | ccaatgaCCCCCTGGCGGCCTA |
|  | Actin-b -HTS -rev | CACGATGGAGGGGAAGACG |
| Untreated- Actin-b | Actin-b -HTS -22-few | ccaatgaCCCCCTGGCGGCCTA |
|  | Actin-b -HTS -rev | TGGGACTGGAGTTGCTTCAT |
| VEGFA+1aa+2aa | VEGFA -HTS -16-few | cagagtcCCAAAGGACCCCAGTCACTC |
|  | VEGFA -HTS -rev | TGGGACTGGAGTTGCTTCAT |
| VEGFA+1aa+2aa | VEGFA -HTS -17-few | cagtactCCAAAGGACCCCAGTCACTC |
|  | VEGFA -HTS -rev | TGGGACTGGAGTTGCTTCAT |
| VEGFA+1aa+2aa | VEGFA -HTS -18-few | cagtctaCCAAAGGACCCCAGTCACTC |
|  | VEGFA -HTS -rev | TGGGACTGGAGTTGCTTCAT |
| VEGFA+1aa+3aa | VEGFA -HTS -18-few | cagtctaCCAAAGGACCCCAGTCACTC |
|  | VEGFA -HTS -rev | TGGGACTGGAGTTGCTTCAT |
| VEGFA+1aa+3aa | VEGFA -HTS -18-few | cagtctaCCAAAGGACCCCAGTCACTC |
|  | VEGFA -HTS -rev | TGGGACTGGAGTTGCTTCAT |
| VEGFA+1aa+3aa | VEGFA -HTS -5-few | caagcttCCAAAGGACCCCAGTCACTC |
|  | VEGFA -HTS -rev | TGGGACTGGAGTTGCTTCAT |
| VEGFA+2aa+4aa | VEGFA -HTS -20-few | cttgagtCCAAAGGACCCCAGTCACTC |
|  | VEGFA -HTS -rev | TGGGACTGGAGTTGCTTCAT |
| VEGFA+2aa+4aa | VEGFA -HTS -20-few | cttgagtCCAAAGGACCCCAGTCACTC |
|  | VEGFA -HTS -rev | TGGGACTGGAGTTGCTTCAT |
| VEGFA+2aa+4aa | VEGFA -HTS -13-few | cactgatCCAAAGGACCCCAGTCACTC |
|  | VEGFA -HTS -rev | TGGGACTGGAGTTGCTTCAT |
| VEGFA+1aa+2aa+3aa | VEGFA -HTS -21-few | ctcacgaCCAAAGGACCCCAGTCACTC |
|  | VEGFA -HTS -rev | TGGGACTGGAGTTGCTTCAT |
| VEGFA+1aa+2aa+3aa | VEGFA -HTS -21-few | ctcacgaCCAAAGGACCCCAGTCACTC |
|  | VEGFA -HTS -rev | TGGGACTGGAGTTGCTTCAT |
| VEGFA+1aa+2aa+3aa | VEGFA -HTS -16-few | cagagtcCCAAAGGACCCCAGTCACTC |
|  | VEGFA -HTS -rev | TGGGACTGGAGTTGCTTCAT |
| VEGFA+1aa+3aa+5aa | VEGFA -HTS -19-few | ctagacaCCAAAGGACCCCAGTCACTC |
|  | VEGFA -HTS -rev | TGGGACTGGAGTTGCTTCAT |
| VEGFA+1aa+3aa+5aa | VEGFA -HTS -19-few | ctagacaCCAAAGGACCCCAGTCACTC |
|  | VEGFA -HTS -rev | TGGGACTGGAGTTGCTTCAT |
| VEGFA+1aa+3aa+5aa | VEGFA -HTS -15-few | cagacatCCAAAGGACCCCAGTCACTC |
|  | VEGFA -HTS -rev | TGGGACTGGAGTTGCTTCAT |
| Untreated- VEGFA | VEGFA -HTS -23-few | cagttacCCAAAGGACCCCAGTCACTC |
|  | VEGFA -HTS -rev | TGGGACTGGAGTTGCTTCAT |
| Untreated- VEGFA | VEGFA -HTS -23-few | cagttacCCAAAGGACCCCAGTCACTC |
|  | VEGFA -HTS -rev | TGGGACTGGAGTTGCTTCAT |
| Untreated- VEGFA | VEGFA -HTS -17-few | caagtagCCAAAGGACCCCAGTCACTC |
|  | VEGFA -HTS -rev | TGGGACTGGAGTTGCTTCAT |
| DNMT1+1aa+2aa | DNMT1 -HTS -10-few | atgtgacACCACACATGTGAACGGACA |
|  | DNMT1 -HTS -rev | CGTGTTCCCCAGAGTGACTT |
| DNMT1+1aa+2aa | DNMT1 -HTS -10-few | atgtgacACCACACATGTGAACGGACA |
|  | DNMT1 -HTS -rev | CGTGTTCCCCAGAGTGACTT |
| DNMT1+1aa+2aa | DNMT1 -HTS -29-few | atgcttcACCACACATGTGAACGGACA |
|  | DNMT1 -HTS -rev | CGTGTTCCCCAGAGTGACTT |
| DNMT1+1aa+3aa | DNMT1 -HTS -11-few | atgcactACCACACATGTGAACGGACA |
|  | DNMT1 -HTS -rev | CGTGTTCCCCAGAGTGACTT |
| DNMT1+1aa+3aa | DNMT1 -HTS -11-few | atgcactACCACACATGTGAACGGACA |
|  | DNMT1 -HTS -rev | CGTGTTCCCCAGAGTGACTT |
| DNMT1+1aa+3aa | DNMT1 -HTS -30-few | atggcgaaCCACACATGTGAACGGACA |
|  | DNMT1 -HTS -rev | CGTGTTCCCCAGAGTGACTT |
| DNMT1+2aa+4aa | DNMT1 -HTS -14-few | cttagctACCACACATGTGAACGGACA |
|  | DNMT1 -HTS -rev | CGTGTTCCCCAGAGTGACTT |
| DNMT1+2aa+4aa | DNMT1 -HTS -14-few | cttagctACCACACATGTGAACGGACA |
|  | DNMT1 -HTS -rev | CGTGTTCCCCAGAGTGACTT |
| DNMT1+2aa+4aa | DNMT1 -HTS -21-few | aagatgcACCACACATGTGAACGGACA |
|  | DNMT1 -HTS -rev | CGTGTTCCCCAGAGTGACTT |
| DNMT1+1aa+2aa+3aa | DNMT1 -HTS -15-few | cttggagACCACACATGTGAACGGACA |
|  | DNMT1 -HTS -rev | CGTGTTCCCCAGAGTGACTT |
| DNMT1+1aa+2aa+3aa | DNMT1 -HTS -15-few | cttggagACCACACATGTGAACGGACA |
|  | DNMT1 -HTS -rev | CGTGTTCCCCAGAGTGACTT |
| DNMT1+1aa+2aa+3aa | DNMT1 -HTS -22-few | aagcgtgACCACACATGTGAACGGACA |
|  | DNMT1 -HTS -rev | CGTGTTCCCCAGAGTGACTT |
| DNMT1+1aa+3aa+5aa | DNMT1 -HTS -13-few | ctagcacACCACACATGTGAACGGACA |
|  | DNMT1 -HTS -rev | CGTGTTCCCCAGAGTGACTT |
| DNMT1+1aa+3aa+5aa | DNMT1 -HTS -13-few | ctagcacACCACACATGTGAACGGACA |
|  | DNMT1 -HTS -rev | CGTGTTCCCCAGAGTGACTT |
| DNMT1+1aa+3aa+5aa | DNMT1 -HTS -20-few | ccaatgaaCCACACATGTGAACGGACA |
|  | DNMT1 -HTS -rev | CGTGTTCCCCAGAGTGACTT |
| Untreated- DNMT1 | DNMT1 -HTS -27-few | atcgaagACCACACATGTGAACGGACA |
|  | DNMT1 -HTS -rev | CGTGTTCCCCAGAGTGACTT |
| Untreated- DNMT1 | DNMT1 -HTS -28-few | atcgtccACCACACATGTGAACGGACA |
|  | DNMT1 -HTS -rev | CGTGTTCCCCAGAGTGACTT |
| Untreated- DNMT1 | DNMT1 -HTS -29-few | atgcttcACCACACATGTGAACGGACA |
|  | DNMT1 -HTS -rev | CGTGTTCCCCAGAGTGACTT |

**Supplementary References**

1. Anzalone AV, Randolph PB, Davis JR, Sousa AA, Koblan LW, Levy JM, et al. Search-and-replace genome editing without double-strand breaks or donor DNA. Nature*.* 2019; 576(7785)**:**149-57.<https://doi.org/10.1038/s41586-019-1711-4>
